# Mapping proteomic composition of excitatory postsynaptic sites in the cerebellar cortex

**DOI:** 10.3389/fnmol.2024.1381534

**Published:** 2024-05-09

**Authors:** Kaylie Robinson, Mathias Delhaye, Ann Marie Craig

**Affiliations:** Djavad Mowafaghian Centre for Brain Health and Department of Psychiatry, University of British Columbia, Vancouver, BC, Canada

**Keywords:** synapse, AMPA, NMDA, Shank, cerebellar glomerulus, expansion microscopy

## Abstract

Functions of the cerebellar cortex, from motor learning to emotion and cognition, depend on the appropriate molecular composition at diverse synapse types. Glutamate receptor distributions have been partially mapped using immunogold electron microscopy. However, information is lacking on the distribution of many other components, such as Shank2, a postsynaptic scaffolding protein whose cerebellar dysfunction is associated with autism spectrum disorders. Here, we used an adapted Magnified Analysis of the Proteome, an expansion microscopy approach, to map multiple glutamate receptors, scaffolding and signaling proteins at single synapse resolution in the cerebellar cortex. Multiple distinct synapse-selective distribution patterns were observed. For example, AMPA receptors were most concentrated at synapses on molecular layer interneurons and at climbing fiber synapses, Shank1 was most concentrated at parallel fiber synapses on Purkinje cells, and Shank2 at both climbing fiber and parallel fiber synapses on Purkinje cells but little on molecular layer interneurons. Our results are consistent with gene expression data but also reveal input-selective targeting within Purkinje cells. In specialized glomerular structures of the granule cell layer, AMPA receptors as well as most other synaptic components preferentially targeted to synapses. However, NMDA receptors and the synaptic GTPase activating protein SynGAP preferentially targeted to extrasynaptic sites. Thus, glomeruli may be considered integrative signaling units through which mossy fibers differentially activate synaptic AMPA and extrasynaptic NMDA receptor complexes. Furthermore, we observed NMDA receptors and SynGAP at adherens junctions, suggesting a role in structural plasticity of glomeruli. Altogether, these data contribute to mapping the cerebellar ‘synaptome’.

## Introduction

**Figure 1:**
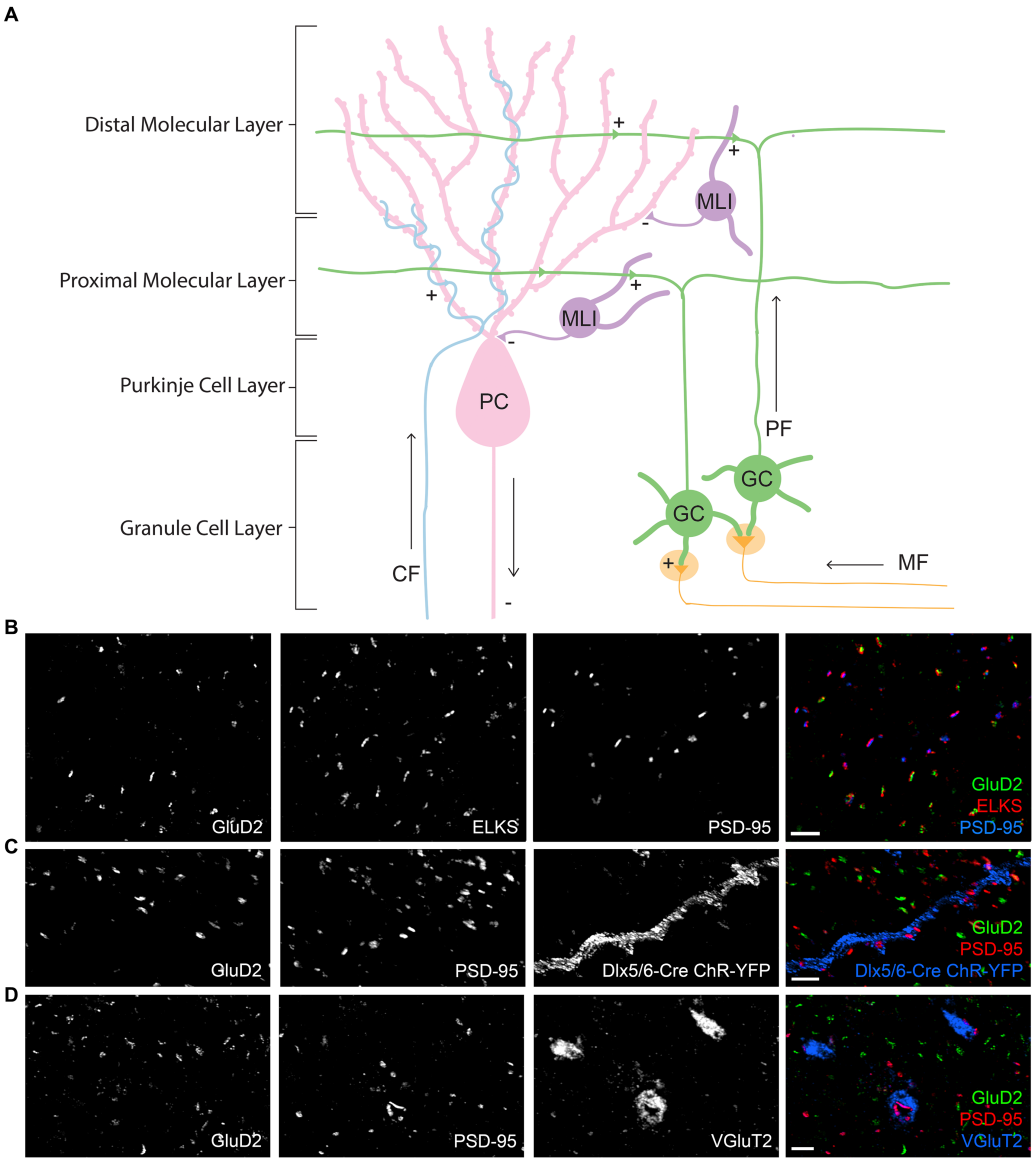
Circuitry and synapse-specific markers in the cerebellar cortex. **(A)** A schematic diagram of simplified circuitry of the cerebellar cortex including the different synapse types and cell types, with ‘+’ indicating excitatory synapses and ‘-‘indicating inhibitory synapses. GC, granule cell; PC, Purkinje cell; MLI, molecular layer interneuron; MF, mossy fiber; PF, parallel fiber; CF, climbing fiber. **(B–D)** Images of expanded mouse cerebellar sections. **(B)** GluD2 and PSD-95 form largely distinct clusters apposing ELKS in the cerebellar molecular layer, at separate synapses. **(C)** Co-staining of GluD2, PSD-95 and GFP (recognizing the genetically encoded ChR2-YFP) in the molecular layer of expanded cerebellum from a *Dlx5/6-Cre Ai32* transgenic mouse shows the presence of strong PSD-95 at PF-MLI synapses along the labeled dendrite. Weaker PSD-95 clusters are present at some PF-PC synapses marked by GluD2. **(D)** Co-staining of GluD2, PSD-95, and VGluT2 in the molecular layer shows the presence of PSD-95, but not GluD2, at VGluT2+ CF synapses. Scale bars, 1 μm biological scale, 3.93 μm expanded scale.

The cerebellum has long been known for its role in fine motor control and balance. A more recent appreciation has developed for broader cerebellar contributions to emotion and cognition and cerebellar dysfunction in autism spectrum disorder (Van Overwalle et al., 2020; van der Heijden et al., 2021; Rudolph et al., 2023). Anatomical connectivity within cerebellar circuits is well understood, particularly for the cerebellar cortex (Figure 1A). Mossy fiber (MF) inputs from multiple brainstem nuclei excite granule cells (GCs) whose parallel fibers (PFs) in turn excite Purkinje cells (PCs), the sole output. PCs are additionally excited by climbing fibers (CFs) from the inferior olive. All cell types are modulated by interneurons, of which the most numerous are molecular layer interneurons (MLIs). MLIs receive inputs from PFs but no direct inputs from CFs. Within this circuitry, synaptic transmission, plasticity, and information processing in relation to motor learning have been intensively studied (Ito, 2006; Hull and Regehr, 2022; Nguyen et al., 2023).

Less is known about the molecular diversity of synapses, how postsynaptic glutamate receptors, scaffolding proteins, and signaling enzymes are differentially distributed to regulate information processing in the cerebellar cortex. Visualizing the spatial distribution of molecular diversity at individual synapses within intact cerebellar circuits has mainly relied on labor-intensive immunogold electron microscopy approaches, as traditional light microscopy approaches lack single synapse resolution.

The focus of synaptic diversity mapping (‘synaptome’) studies in the cerebellum has been on the primary functional components, the glutamate receptors. In the molecular layer, immunogold electron microscopy was used to reveal a higher density of AMPA receptors at CF-PC and PF-MLI synapses than at PF-PC synapses (Masugi-Tokita et al., 2007; Yamasaki et al., 2011). This biased distribution of AMPA receptors is also reflected in differences in electrophysiological measures (Barbour, 1993; Carter and Regehr, 2002; Foster et al., 2002). NMDA receptors are sparsely expressed in the molecular layer, contributing a minor component to transmission at CF-PC but not PF-PC synapses (Piochon et al., 2007), and present only at extrasynaptic sites on MLIs where their activation by spillover is critical for some forms of plasticity and motor learning (Clark and Cull-Candy, 2002; Kono et al., 2019). At MF-GC synapses, AMPA receptors are exclusively postsynaptic, yet the close spacing between synapses and confined space within the glomerular structures facilitates their activation by spillover as well as by direct release (DiGregorio et al., 2002). NMDA receptors on GCs play a key role in transmission and integration through the GC layer and are essential for long term potentiation at MF-GC synapses and vestibulo-cerebellar motor learning (D'Angelo et al., 1999; Andreescu et al., 2011; Schwartz et al., 2012). However, there is controversy regarding the distribution of NMDA receptors on GCs, whether they are located at postsynaptic sites (Yamada et al., 2001; Abe et al., 2004; Ito, 2006) or at extrasynaptic sites (Petralia et al., 2002; Cathala et al., 2003).

Beyond these glutamate receptors, less information is available about the distribution of other excitatory postsynaptic molecules in the cerebellar cortex. GluD2, an atypical receptor that functions as a synaptic organizing protein, is specifically localized to PF-PC synapses (Landsend et al., 1997). Neuroligin-1 is selectively concentrated at PF-MLI synapses and the scaffolding protein PSD-95 is more concentrated at PF-MLI and CF-PC than PF-PC synapses (Yamasaki et al., 2011; Nozawa et al., 2018). PC-specific deletion of another scaffolding protein, Shank2, alters synapse density, PC spiking and plasticity, and generates autism-like behavior, supporting its functional importance (Ha et al., 2016; Peter et al., 2016). Shank1-3 are all high risk genes for neurodevelopmental disorders (Monteiro and Feng, 2017; Wan et al., 2022) and are expressed in the cerebellum. Yet cerebellar synaptome mapping information is not available for Shank1-3 or many other postsynaptic proteins.

Here, we use an adapted version of Magnified Analysis of the Proteome [MAP; (Ku et al., 2016; Delhaye et al., 2024)] to map the distributions of multiple excitatory postsynaptic proteins in the cerebellar cortex with single synapse resolution. MAP employs immunolabeling after tissue clearing and expansion to circumvent limitations of antibody accessibility and of light microscopy resolution. Our results confirm previous findings for AMPA receptor distributions and reveal differential distributions of Shank1-3 among synapse types. Furthermore, in the glomerular structure containing tightly packed MF-GC synapses where the advantages of the MAP approach are critical, our data reveal a surprising selective localization of NMDA receptor complexes to extrasynaptic sites including adherens junctions.

## Materials and methods

### Animals

Animal experiments were done following ethical guidelines of the Canadian Council on Animal Care as well as institutional requirements of the University of British Columbia. Experiments were conducted on adult male and female mice at least 6 months old. These animals had unrestricted access to food and water and were housed on a 12 h light/dark cycle. Although under-powered, statistical analysis of males versus females showed no significant differences, so they were grouped together during analysis. Wild type mice were C57BL/6 J (Jackson Laboratory) and were used for the majority of the study. The mice with a subset of GABAergic interneurons labeled with ChR2-EYFP (Figure 1B) were Dlx5/6-Cre (Tg(dlx5a-cre)1Mekk; RRID:IMSR_JAX:008199) crossed with Ai32 *Gt(ROSA)26Sor^tm32(CAG-COP4∗H134R/EYFP)Hze^* (RRID:IMSR_JAX:012569) mice and were maintained on a C57BL/6 J background (Zerucha et al., 2000; Madisen et al., 2015; Luo et al., 2020).

### Antibodies

Primary antibodies used include: ELKS (mouse IgG2a, Sigma, Cat: E4531, RRID: AB_2100013, 1:250; recognizes all ELKS1α and ELKS2α isoforms which are the major isoforms (Liu et al., 2014)), GFP (rabbit, Thermofisher, Cat: A11122, RRID: AB_221569, 1:200), GluA2 (guinea pig, Synaptic Systems, Cat: 182105, RRID: AB_2619875, 1:250), GluN1 (mouse IgG2b, Synaptic Systems, Cat: 114011, RRID: AB_887750, 1:250), GluD2 (guinea pig, Nittobo Medical, Cat MSFR102600, RRID: AB_2571603, 1:200), GluD2 (rabbit, Novus, Cat: NBP2-31632, 1:400), M-cadherin (mouse IgG1, Santa Cruz, Cat: sc-81471, RRID: AB_2077111, 1:25), panAMPA (guinea pig, Nittobo Medical, Cat: MSFR104670, RRID: AB_2571610, 1:250), panMAGUK (mouse IgG1, Antibodies Inc., Cat: 75–029, RRID: AB_2877192, 1:250), PSD-95 (mouse IgG1, Synaptic Systems, Cat: 124011, RRID: AB_10804286, 1:250), PSD-95 (mouse IgG2a, NeuroMab, Cat: 75–028, Clone: K28/43, RRID: AB_2877189, 1:50), RIM1/2 (guinea pig, Synaptic Systems, Cat: 140205, RRID: AB_2631216, 1:200; recognizes RIM1 and RIM2), SAPAP1 (guinea pig, Synaptic Systems, Cat: 342104, RRID: AB_2620088, 1:200), Shank1 (chicken, Synaptic Systems, Cat: 162106, RRID: AB_2832227, 1:200), Shank2 (guinea pig, Synaptic Systems, Cat: 162204, RRID: AB_2619861, 1:200), Shank3 (guinea pig, Synaptic Systems, Cat: 162304, RRID: AB_2619863, 1:200), SynGAP (rabbit, Thermofisher, Cat: PA1-046, RRID: AB_2287112, 1:250), VGluT2 (mouse IgG2a, Synaptic Systems, Cat: 135421, RRID: AB_2619823, 1:200). All secondary antibodies were goat antibodies, which include: anti-rabbit Alexa 488 (Invitrogen, Cat: A32731), anti-mouse IgG2b Alexa 488 (Thermofisher Life Technologies, Cat: A21141), anti-chicken Alexa 488 (Invitrogen, Cat: A32931), anti-guinea pig Alexa 488 (Invitrogen, Cat: A11073), anti-rabbit Alexa 568 (Invitrogen, Cat: A11036), anti-mouse IgG1 Alexa 568 (Invitrogen, Cat: A21124), anti-mouse IgG2a Alexa 568 (Invitrogen, Cat: A21245), anti-guinea pig Alexa 568 (Invitrogen, Cat: A11075), anti-mouse IgG2a Alexa 647 (Invitrogen, Cat: A21241), anti-mouse IgG1 Alexa 647 (Thermofisher Life Technologies, Cat: A21240).

### Adapted MAP procedure

The MAP procedure was adapted from the original (Ku et al., 2016) with slight modifications (Delhaye et al. (2024); this reference includes a detailed step by step protocol).

Adult mice were anesthetized with urethane and perfused transcardially with Ringer’s solution containing heparin followed by fixation solution (4% PFA in PBS). The brain was extracted and placed in fixation solution overnight at 4°C, then for 2 h at RT. All incubations were done with gentle shaking unless otherwise stated. After rinsing the brain in washing solution (0.02% NaN_3_ (Sigma, S2002) in PBS), the cerebellum was removed and the entire cerebellum was sectioned sagittally using a vibratome (thickness 170 μm). Sections were then incubated overnight in fixation solution at 4°C, then moved to 37°C for 2 h.

Following further incubations with washing solution, sections were stained with DAPI (1:30000 in PBS) for 30 min. Pre-expansion images were acquired using a Zeiss Axiozoom microscope at 40x magnification. Sections were then incubated in low-AA solution (4% Acrylamide (Sigma, A3553), 4% PFA in PBS) at 4°C overnight, then for 2 h at RT. After further washing, sections were incubated in inactivation solution (1% acetamide (Sigma, A0500), 1% glycine (Sigma, G7126), 0.02% NaN_3_, pH 9.0) for 4 h at 37°C, then washed again before being incubated in MAP solution (30% Acrylamide, 10% Sodium Acrylate (Sigma, 408220), 0.1% Bisacrylamide (Bio-rad, 161-0142), 0.03% V-50 (Sigma, 440914) in PBS) at 4°C overnight.

For gel embedding, sections were taken out of the MAP solution and placed into a home-made gelation chamber with fresh MAP solution and sealed with a coverslip. All gelation chambers were placed into a gelation box and the air purged with nitrogen. The gelation box was incubated at 45°C for 2 h, no shaking. Gel-tissue hybrid sections were removed from the gelation chambers, excess gel was trimmed from around the sections, and they were placed in washing solution at 4°C for up to two weeks, without shaking.

For denaturation, clearing and expansion, sections were incubated in denaturation solution (200 mM SDS, 200 mM NaCl, 50 mM of Tris, pH 9) at 37°C for 2 h then at 95°C for 45 min. The sections were then washed twice with 0.001x PBS for 1 h each at RT, then overnight in 0.001x PBS (0.001x PBS is PBS diluted 1:1000 in water; the low salt expands the sample while washing out the denaturation solution). Before immunostaining, samples were incubated in PBS-T (0.1% Triton-X100 in PBS) at RT for 30 min to equilibrate the samples in the appropriate buffer for antibody staining.

Sections were randomly selected and stained with primary antibodies at the concentrations listed above for 48 h in PBS-T at 4°C. Sections were then washed at 37°C in PBS-T before being incubated in secondary antibodies at a 1:200 concentration for 48 h in PBS-T at 4°C. Following secondary antibodies, the sections were washed in PBS-T at RT, then stained with DAPI at a 1:30000 concentration for 1 h at RT in PBS-T before another wash in PBS-T. Following this, the solution was changed to 0.001x PBS and the sections were incubated overnight at RT.

### Imaging

For imaging, the expanded sections were mounted on poly-L-lysine-coated slides in custom printed imaging chambers filled with 0.001x PBS. The imaging chamber 3D printing code is available at: https://osf.io/w6c9u/. Post-expansion DAPI images were taken on a Zeiss Axiozoom microscope at 16x magnification, for calculating the expansion factor by comparison with the pre-expansion DAPI images. High resolution image stacks (53 μm × 53 μm × 7.41 μm with a voxel size of 50 nm × 50 nm × 190 nm) were acquired using a Zeiss LSM 980 Airyscan microscope, with the 1.2 numerical aperture objective LD LCI Plan-Apochromat 40x/1.2 Imm Corr DIC M27. The super-resolution mode was used, and these images were directly processed using the Airyscan processing from Zeiss. For each different co-stain for quantitative analysis, 2 image stacks were taken from randomly selected regions for each section, and 2 sections were taken for each antigen of interest, from 3 separate mice.

### Analysis

#### Expansion factors

DAPI images were taken after the post-fixation step, before the samples were expanded, on a Zeiss AxioZoom Macroscope at 40x zoom, as well as after expansion and immunostaining on the same Zeiss AxioZoom Macroscope at 16x zoom. Images before and after expansion were then overlayed in Adobe Photoshop and an expansion factor was calculated. For the density calculations in the Supplementary Figures and scale bars, expansion factors were calculated for 20 random samples and an average expansion factor used.

#### Signal quantification

All signal quantification was done in 3D using Arivis Vision 4D. The entirety of each image stack was selected as the region of interest. Channels were then thresholded for intensity to define clusters and for minimal size to exclude pixel noise. For the molecular layer, a simple intensity threshold was used. For the granule cell layer, a watershed algorithm was used to define clusters. Any clusters touching the edge of the stack were excluded, so as to measure only complete synapses. PF-PC synapses were defined by the presence of GluD2, while nonPF-PC synapses were defined by the presence of PSD-95 and absence of GluD2. Synapses were considered positive for an antigen of interest if the antigen was within 300 nm (~75 nm biological scale) of GluD2 or PSD-95. CF synapses were defined as synapses that were PSD-95 and VGluT2 positive, and nonCF synapses were defined as synapses that were PSD-95 positive and VGluT2 negative. Synapses were considered antigen positive if the antigen was within 300 nm (~75 nm biological scale) of VGluT2 or PSD-95. In the granule cell layer, excitatory synapses were defined by the presence of PSD-95 and ELKS and were considered positive for the antigen of interest if the antigen was within 300 nm (~75 nm biological scale) of ELKS and PSD-95. Extrasynaptic clusters were defined by the presence of PSD-95 and absence of ELKS and were considered antigen positive if the antigen was within 30 nm (~7.5 nm biological scale) of PSD-95. For every staining combination, integrated intensity per cluster and density measures were exported for each stack.

#### Data processing

Data analysis was performed using Excel and R studio. Image stacks taken for the proximal and distal regions of the molecular layer were analyzed separately. All intensity values were normalized to the mean of each measure for each stack. Clusters were then separated into their respective synapse type, or synaptic and extrasynaptic in the case of the granule cell layer. For each stack, measures were made for cluster density per tissue volume, and for integrated intensity per cluster. Calculations for percent antigen positive and ratio measures were then performed for each stack. PF-PC synapses were further separated into PSD-95+ and PSD-95- and similar measures made for each stack. Values for each measure for all stacks from one mouse were then averaged. Each data point represents one mouse for *n* = 3 mice (or > 3 mice for measures of PSD-95 or GluD2 that were co-stained with all antigens).

#### Statistics

All statistical analysis was performed in GraphPad Prism and R studio. One-way, two-way or three-way ANOVA was used depending on the number of variables in the experiment, with appropriate post-hoc comparisons. Statistical details along with main *p* values are provided in the figure legends. A list of *p* values for post-hoc comparisons is provided in Supplementary Table S1.

## Results

### Selection of synapse-specific markers and proteins of interest

To assess synaptic protein distributions in the mouse cerebellum molecular layer, we first validated synapse-specific markers for co-staining. We used GluD2 to label PF-PC synapses as GluD2 is localized only to this synapse type (Landsend et al., 1997). While we are not aware of a marker specific for PF-MLI synapses, we noted that PSD-95 has a distribution largely distinct from that of GluD2, with both PSD-95 and GluD2 apposed to the presynaptic component ELKS (Figure 1B). Indeed, PSD-95 was previously found to be more concentrated at PF-MLI and CF-PC than PF-PC synapses (Yamasaki et al., 2011; Nozawa et al., 2018). We confirmed the presence of bright PSD-95 clusters at PF-MLI synapses using a genetic label for a subset of MLIs (Figure 1C). This genetic label was generated by crossing Dlx5/6-Cre (Tg(dlx5a-cre)1Mekk) with Ai32 *Gt(ROSA)26Sor^tm32(CAG-COP4∗H134R/EYFP)Hze^* mice, resulting in ChR2-EYFP expression in all GABAergic interneurons in forebrain and in a subset of such neurons in cerebellum (Zerucha et al., 2000; Madisen et al., 2015; Luo et al., 2020). The vesicular glutamate transporter VGluT2 is a well-accepted marker for CF-PC terminals (Fremeau et al., 2001), which we also confirmed have PSD-95+ GluD2- postsynaptic sites (Figure 1D). Thus, PF-PC synapses were defined as being GluD2+ (of which a subset are PSD-95+, Figure 1B), PF-MLI synapses were defined as being GluD2- PSD-95+ VGluT2-, and CF-PC synapses were defined as being GluD2- PSD-95+ VGluT2+. Unfortunately, with only 3 imaging channels available, we could not co-stain for each protein of interest with GluD2, PSD-95, and VGluT2 all together in one experiment. Thus we first co-stained for each protein of interest with GluD2 and PSD-95 to classify synapses as PF-PC (GluD2+) and nonPF-PC (GluD2- PSD-95+ corresponding to PF-MLI and CF-PC synapses). Then we co-stained for each protein of interest with VGluT2 and PSD-95 to classify synapses as CF-PC (PSD-95+ VGluT2+) and nonCF-PC (PSD-95+ VGluT2- corresponding to PF-MLI and some PF-PC synapses).

We chose key postsynaptic glutamate receptor subunits, scaffolding proteins, and enzymes for mapping synaptic diversity in the cerebellar molecular layer. To assess glutamate receptors, we used a panAMPA antibody that recognizes all AMPA receptor subunits, an antibody against the major AMPA receptor subunit GluA2, and an antibody against the essential NMDA receptor subunit GluN1. We chose Shank1-3 for assessment because of their importance in neurodevelopmental disorders as discussed in the introduction. We assessed another excitatory postsynaptic scaffold protein, SAPAP1 (also known as DLGAP1, DAP1 and GKAP), which functions to link Shank proteins to PSD-95 (Naisbitt et al., 1999; Bai et al., 2022). Mutations in the human gene encoding SAPAP1 are also associated with neuropsychiatric disorders, including a *de novo* CNV in schizophrenia (Kirov et al., 2012; Bai et al., 2022). Finally, we assessed the enzyme SynGAP, a postsynaptic GTPase-activating protein for Ras and Rap and one of the strongest single gene risk factors for intellectual disability (Gamache et al., 2020). Although SynGAP is most abundant in the forebrain, it is expressed at a lower level in the cerebellar molecular layer (Lein et al., 2007; Moon et al., 2008; Kozareva et al., 2021).

### Synaptic proteins are differentially distributed among PF-PC and nonPF-PC synapses in the molecular layer

**Figure 2:**
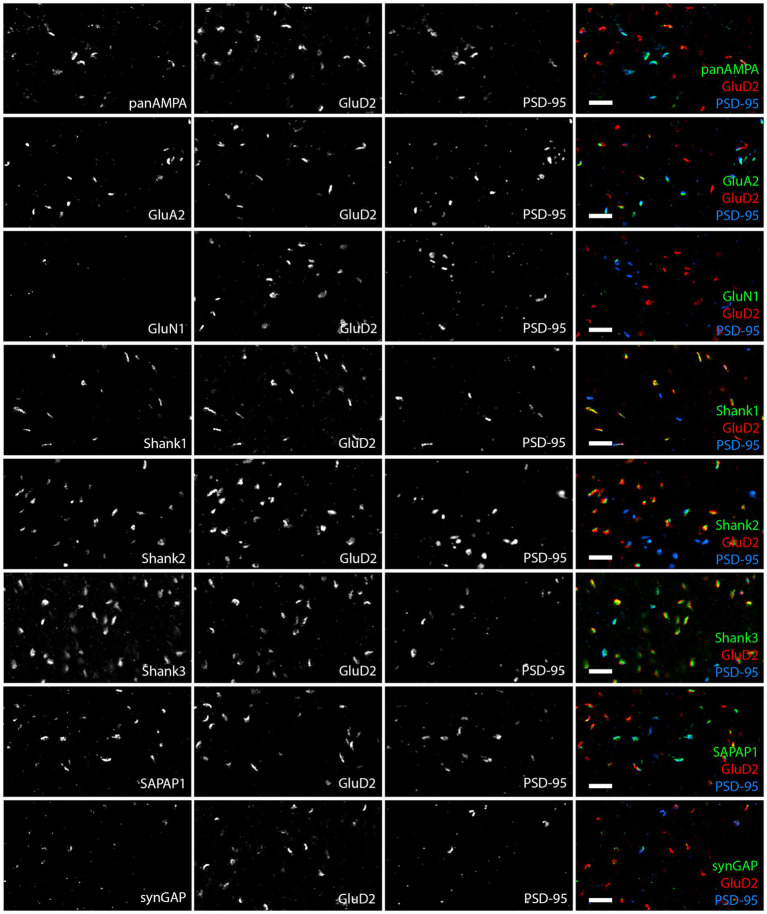
Diversity in synaptic composition of PF-PC versus nonPF-PC synapses. Images of expanded mouse cerebellar sections taken in the proximal molecular layer region showing each antigen of interest co-stained with GluD2 and PSD-95. GluD2 marks PF-PC synapses while PSD-95 marks mainly nonPF-PC excitatory synapses and is also detected at some PF-PC synapses. Synaptic components exhibited different distribution patterns, with panAMPA, GluA2, and SAPAP1 higher at nonPF-PC synapses, Shank1 and Shank2 higher at PF-PC synapses, Shank3 and lower levels of SynGAP present at both synapse types, and GluN1 detected at very few synapses. Scale bars, 1 μm biological scale, 3.93 μm expanded scale.

**Figure 3:**
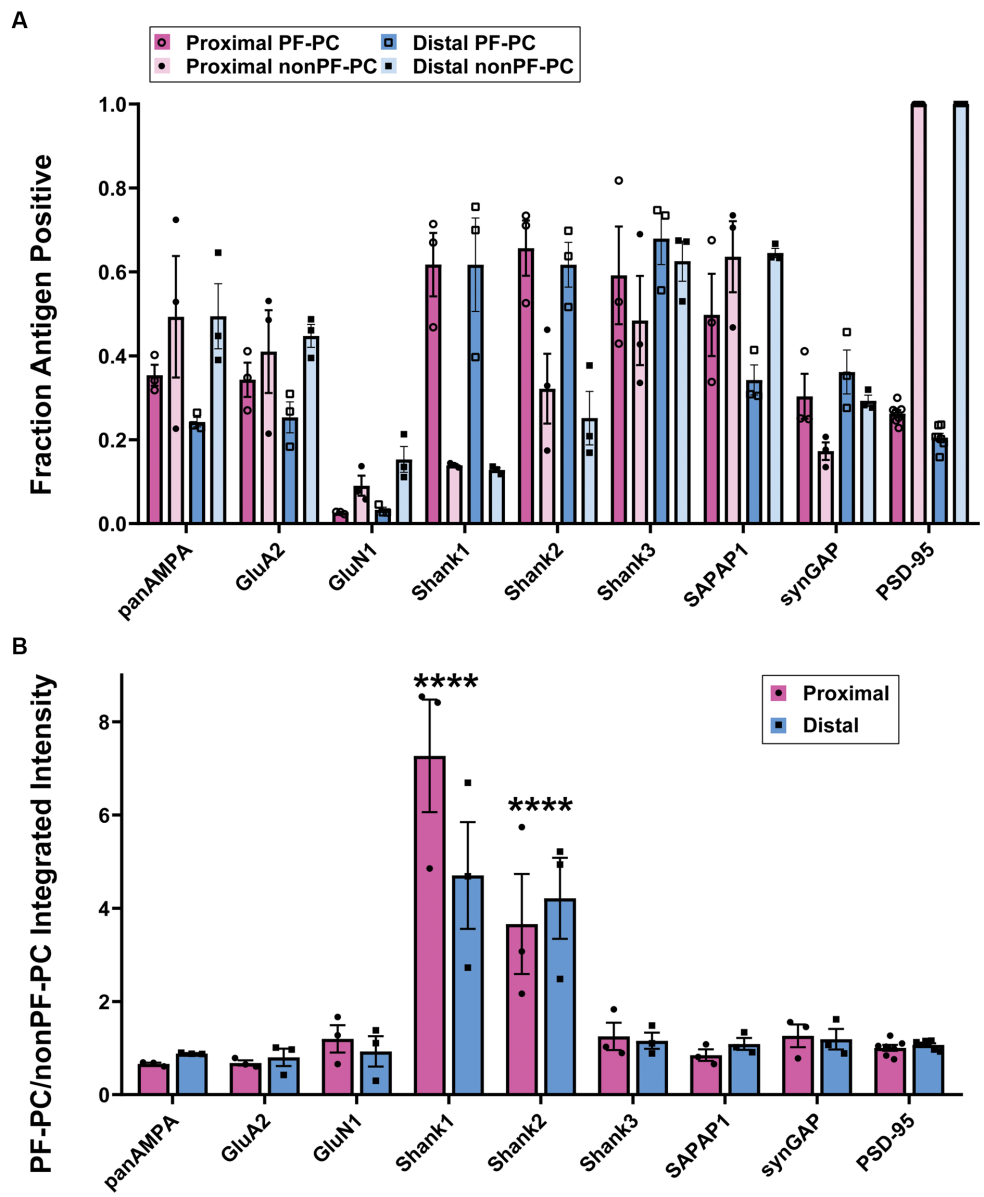
Quantitation of differential synaptic composition at PF-PC versus nonPF-PC synapses. Brains were processed using the MAP procedure and each section was stained with an antigen of interest along with GluD2 and PSD-95, as in Figure 2. Two image stacks were taken from the proximal region of the molecular layer and two from the distal region per section, with two sections from each mouse, and a total of three mice. Values from each mouse were averaged (*n* = 3 mice). **(A)** Fraction of PF-PC and nonPF-PC synapses positive for each antigen. 3-way ANOVA showed significant differences with antigen and PF-PC vs. nonPF-PC but not Proximal vs. Distal: Antigen *p* < 0.0001, PF-PC vs. nonPF-PC *p* = 0.017, Proximal vs. Distal *p* = 0.975, Antigen x PF-PC vs. nonPF-PC *p* < 0.0001, Antigen x Proximal vs. Distal *p* = 0.183, PF-PC vs. nonPF-PC x Proximal vs. Distal *p* = 0.070, Antigen x PF-PC vs. nonPF-PC x Proximal vs. Distal x *p* = 0.958. *p*-values for Tukey’s post-hoc comparisons can be found in Supplementary Table S1. **(B)** Ratio of integrated intensity per synapse at antigen-positive PF-PC/nonPF-PC synapses for each antigen of interest in proximal and distal regions of the molecular layer. 2-way ANOVA showed a significant difference between antigens but not between proximal and distal, with antigen *p* < 0.0001, Proximal vs. Distal *p* = 0.372, interaction *p* = 0.102. Sidak’s post-hoc comparisons showed significant differences in Shank1 (****p < 0.0001) and Shank2 (****p < 0.0001) when compared with all other antigens, no other significant differences were shown, all *p* values can be found in Supplementary Table S1.

We proceeded to co-stain each protein of interest with GluD2 and PSD-95 and acquired high resolution image stacks in the proximal and distal molecular layer with a Zeiss LSM 980 using the Airyscan (Figure 2). Synapses were classified as PF-PC (GluD2+) and nonPF-PC (GluD2- PSD-95+). Taking into account subsequent experiments using VGluT2 (Supplementary Figure S3), we estimate that the majority of these nonPF-PC synapses are PF-MLI with up to 20% being CF-PC synapses. Shank1 and Shank2 appeared more concentrated at PF-PC synapses whereas panAMPA, GluA2, and SAPAP1 appeared more concentrated at nonPF-PC synapses. Shank3 appeared abundant and SynGAP present at lower levels at both synapse types, while little GluN1 was detected at either synapse. Quantitative measures in 3D confirmed these impressions, revealing significant differences among proteins and between synapse types in all measures (Figure 3; Supplementary Figure S1; statistical tests with main *p* values are reported in the figure legends and *p* values for post-hoc tests are reported in Supplementary Table S1). The fraction of synapses positive for Shank1 and Shank2 was higher for PF-PC than nonPF-PC synapses (Figure 3A) and at the positive synapses the integrated intensity reflecting the amount of protein per synapse was higher by mean 6.0- and 3.9-fold at PF-PC than nonPF-PC synapses for Shank1 and Shank2, respectively (Figure 3B). In contrast, the fraction of synapses positive for SAPAP1 was higher for nonPF-PC than PF-PC synapses with a similar trend seen for panAMPA (Figure 3A). The amount per positive synapse was higher for panAMPA and GluA2 at nonPF-PC than at PF-PC synapses (Supplementary Figure S1B). The density measures (Supplementary Figure S1A) also revealed differences in the relative numbers of each synapse type in the regions imaged. PF-PC synapses (GluD2 measures for PF-PC synapses, as GluD2 was at all of these synapses) were mean 2.3-fold more abundant than nonPF-PC synapses (PSD-95 measures for nonPF-PC synapses, as PSD-95 was at all of these synapses). Perhaps surprisingly, our analyses did not reveal any significant difference in any measure between proximal and distal molecular layers (Figure 3; Supplementary Figure S1).

**Figure 4:**
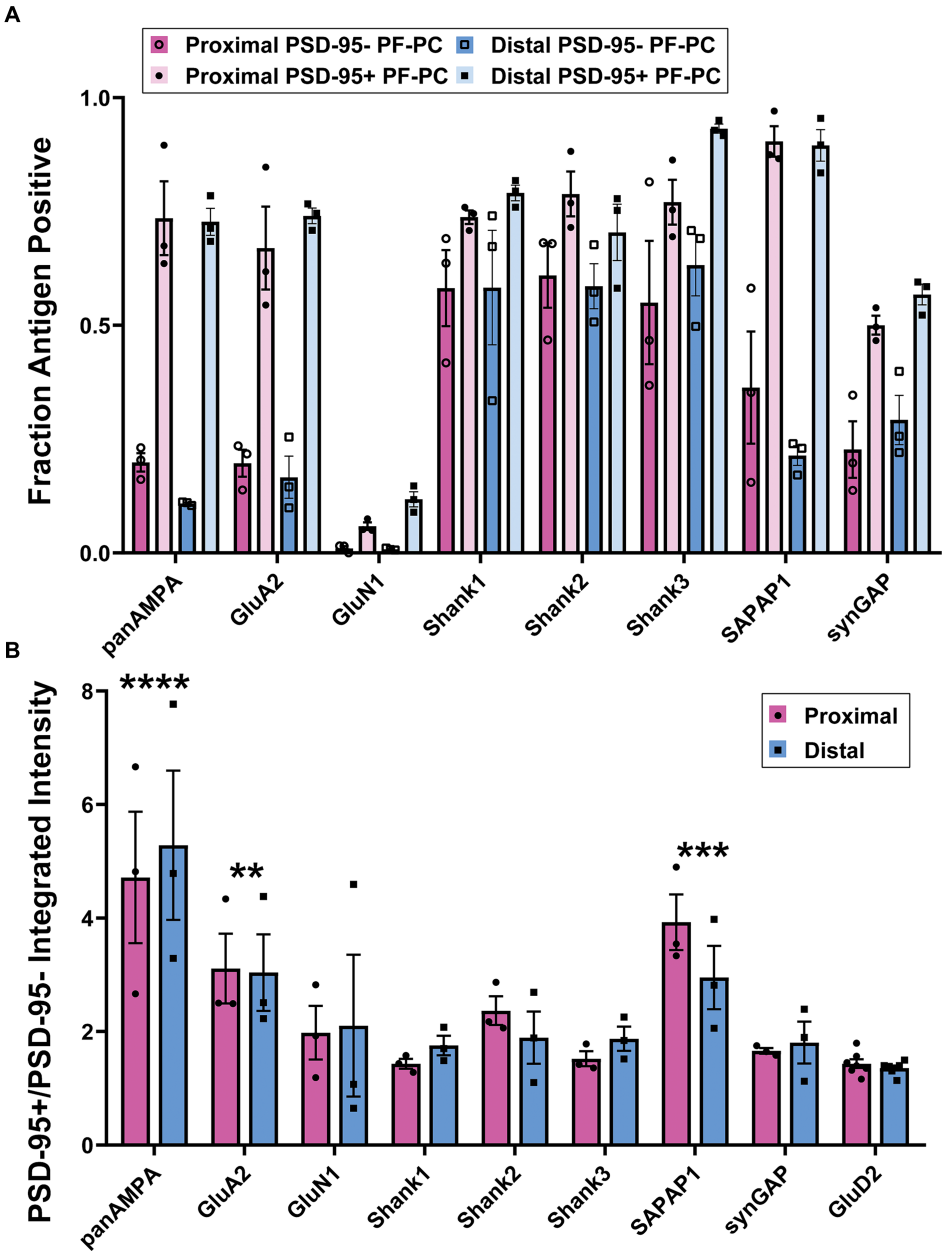
Quantitation of differential synaptic composition at PSD-95+ versus PSD-95- PF-PC synapses. Brains were processed and imaged as in Figures 2, 3. PF-PC synapses were separated into categories according to the presence or absence of detectable PSD-95 and assessed for other synaptic components. **(A)** Fraction of PSD-95+ PF-PC and PSD-95- PF-PC synapses positive for each antigen. 3-way ANOVA showed significant differences with antigen and PSD-95+ vs. PSD-95- but not Proximal vs. Distal: Antigen *p* < 0.0001, PSD-95+ vs. PSD-95- *p* < 0.0001, Proximal vs. Distal *p* = 0.622, Antigen x PSD-95+ vs. PSD-95- < 0.0001, Antigen x Proximal vs. Distal *p* = 0.242, Proximal vs. Distal x PSD-95+ vs. PSD-95- *p* = 0.168, Antigen x Proximal vs. Distal x PSD-95+ vs. PSD-95- *p* = 0.958 (*n* = 3 mice). *p*-values for Tukey’s post-hoc comparisons can be found in Supplementary Table S1. **(B)** Ratio of integrated intensity per synapse at antigen-positive PSD-95+/PSD-95- PF-PC synapses for each antigen of interest in proximal and distal regions of the molecular layer. 2-way ANOVA showed significant differences between antigens but not between proximal and distal, with antigen *p* < 0.0001, Proximal vs. Distal *p* = 0.969, interaction *p* = 0.936. Sidak’s post-hoc comparisons show that panAMPA (*p* < 0.0001), GluA2 (*p* = 0.007) and SAPAP1 (*p* = 0.0006) are significantly different compared with GluD2, where the other antigens are not (GluN1 *p* = 0.719, Shank1 *p* = 0.999, Shank2 *p* = 0.582, Shank3 *p* = 0.994, SynGAP *p* = 0.989).

We wondered whether there is a difference between the PF-PC synapses containing PSD-95 clusters (23%) and those lacking detectable PSD-95 with respect to any of the other proteins of interest so we compared these synapse populations (Figure 4; Supplementary Figure S2). The fraction positive was greater for panAMPA, GluA2, SAPAP1, Shank3, and SynGAP at PSD-95+ relative to PSD-95- PF-PC synapses (Figure 4A). Furthermore, the amount of protein per positive synapse was greater for all nine proteins assessed at PSD-95+ PF-PC compared with PSD-95- PF-PC synapses (Supplementary Figure S2B). These findings suggest that PSD-95+ PF-PC synapses may be larger than the PSD-95- PF-PC synapses, with all antigens more readily detected. Indeed, the amount of panAMPA and GluD2 per synapse was previously found to correlate with the size of PF-PC synapses by immunogold electron microscopy (Masugi-Tokita et al., 2007). In our measures, panAMPA, GluA2, and SAPAP1 also differed from the other proteins in having a relatively greater amount of protein per positive synapse (Figure 4B) for PSD-95+ relative to PSD-95- PF-PC synapses. Similar to the findings above, there was no significant difference between proximal versus distal molecular layer for any of these measures (Figure 4; Supplementary Figure S2).

Overall, these results showed that there are significant differences in the distribution of the proteins of interest at PF-PC versus nonPF-PC as well as at PSD-95+ versus PSD-95- PF-PC synapses. Shank1 and Shank2 are selectively concentrated at PF-PC synapses while panAMPA, GluA2, and SAPAP1 are selectively concentrated at nonPF-PC synapses. Shank3 and SynGAP are more uniformly distributed to both synapse types, and GluN1 poorly expressed at either. Among PF-PC synapses, PSD-95 appears to mark the larger synapses which also have higher concentrations of panAMPA, GluA2, and SAPAP1.

### Synaptic proteins are differentially distributed among CF-PC and nonCF-PC synapses in the molecular layer

**Figure 5:**
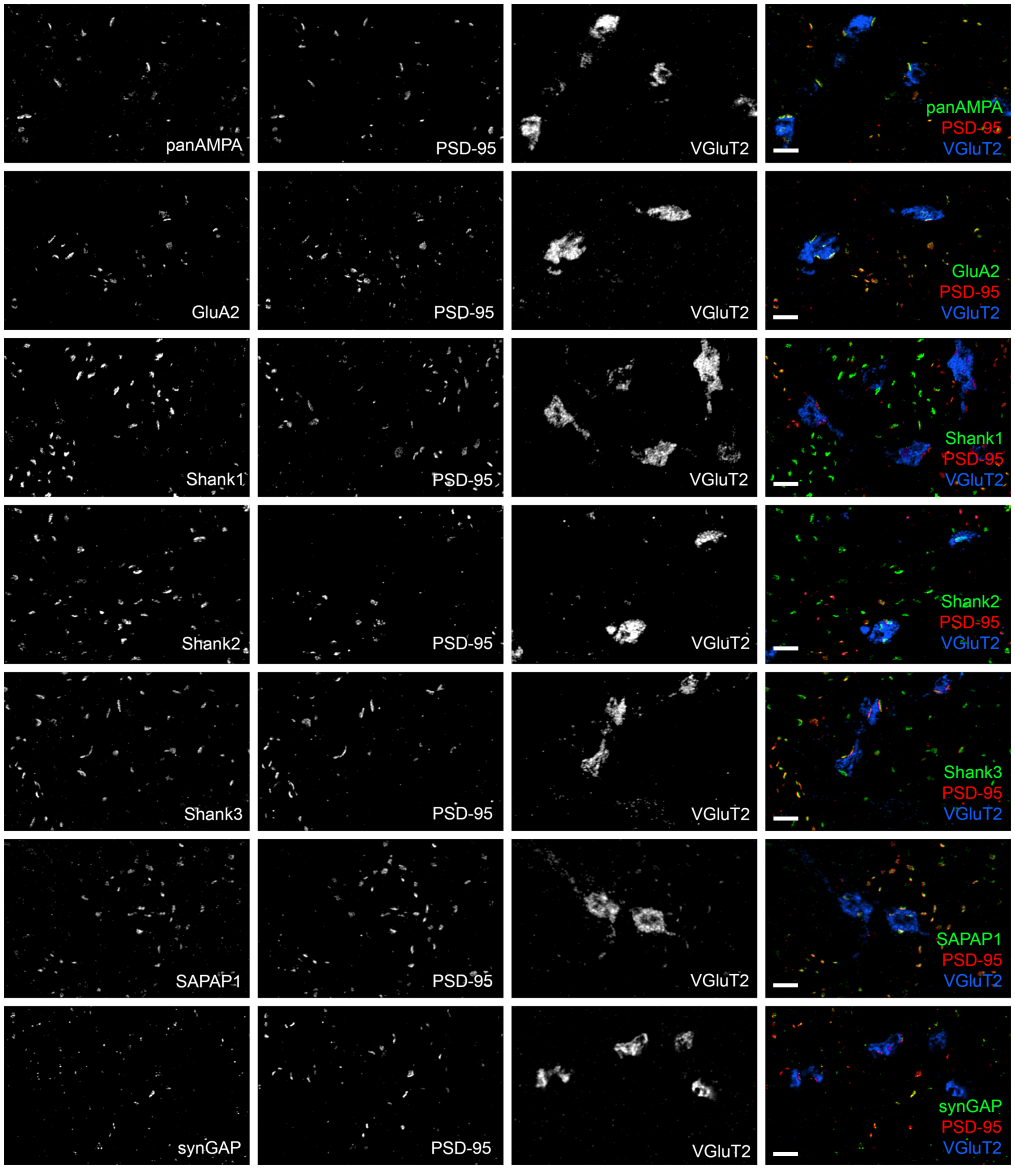
Diversity in synaptic composition of CF-PC versus nonCF-PC synapses. Images of expanded mouse cerebellar sections taken in the molecular layer region showing each antigen of interest co-stained with PSD-95 and VGluT2. VGluT2 marks presynaptic CF terminals. PSD-95 clusters adjacent to VGluT2 clusters were considered CF-PC synapses, while PSD-95 clusters without VGluT2 are nonCF-PC synapses, mainly PF-MLI and some PF-PC synapses. There are additional PSD-95-lacking PF-PC synapses in the fields of view, where Shank proteins are prominent. Many synaptic components showed a similar distribution between CF-PC and nonCF-PC PSD-95-positive synapses; however, Shank1 and SynGAP appeared to be relatively higher at some nonCF-PC synapses. Scale bars 1 μm biological scale, 4.09 μm expanded scale.

**Figure 6:**
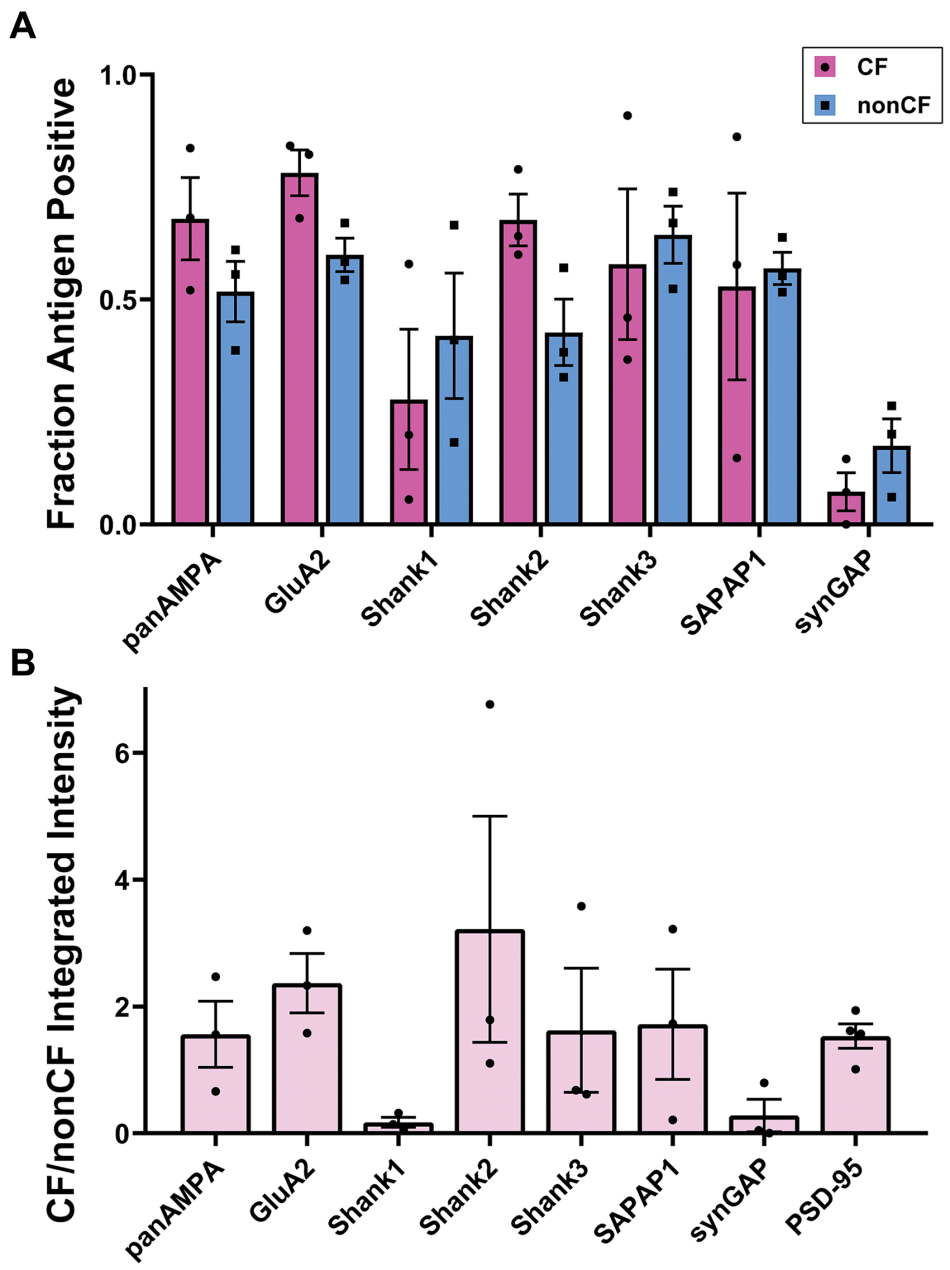
Quantitation of synaptic composition at CF-PC versus nonCF-PC synapses. Brains were processed using the MAP procedure and each section was stained with an antigen of interest along with PSD-95 and VGluT2, as in Figure 5. Two image stacks were taken from the proximal region of the molecular layer per section, with two sections from each mouse, and a total of three mice. Values from each mouse were averaged (*n* = 3 mice). **(A)** Fraction of PSD-95-positive CF-PC and nonCF-PC synapses positive for each antigen. 2 way ANOVA showed significant differences with antigen, but not CF-PC vs. nonCF-PC: Antigen *p* < 0.0001, CF-PC vs. nonCF-PC *p* = 0.535, interaction *p* = 0.357. **(B)** Ratio of integrated intensity per synapse at antigen-positive PSD-95-positive CF-PC/nonCF-PC synapses for each antigen of interest. One-way ANOVA did not show significance: *p* = 0.221.

The same proteins of interest were assessed at PSD-95-positive CF-PC versus nonCF-PC synapses in the molecular layer, excluding GluN1 due to its extremely low expression. Each protein was co-stained with PSD-95 and VGluT2, a component specific to CF terminals, and image stacks acquired in regions with abundant VGluT2 (Figure 5). CF-PC synapses were defined as VGluT2+ PSD-95+ [CFs do not make direct synapses onto MLIs (Szapiro and Barbour, 2007)] and nonCF-PC synapses as VGluT2- PSD-95+. These nonCF-PC synapses correspond to PF-MLI synapses and the PSD-95+ PF-PC synapses, as discussed earlier, with each type contributing about half of the total (based on the PSD-95 measures in Supplementary Figure S1A). While most proteins of interest were readily detected at both CF-PC and nonCF-PC synapses, Shank1 and SynGAP appeared enriched at nonCF-PC synapses with little detected at CF-PC synapses (Figure 5). Quantitative measures supported these observations (Figure 6; Supplementary Figure S3), although in some cases by trends rather than statistically significant differences. The low density of CF-PC synapses (Supplementary Figure S3A) and thus low numbers may have resulted in poor accuracy and high variability in the measures. Nonetheless, Shank1 and SynGAP showed a significantly lower density of positive CF-PC synapses compared with the other proteins (Supplementary Figure S3A) and showed a trend toward a correspondingly lower fraction of CF-PC compared with nonCF-PC synapses (Figure 6A). Furthermore, at the positive synapses, the amount of Shank1 and SynGAP showed a trend toward being lower at CF-PC synapses, with mean values only 6 and 28%, respectively, of the amount at nonCF-PC synapses (Figure 6B; Supplementary Figure S3C). At the opposite end of the spectrum, the amount of Shank2 showed a trend to be higher at CF-PC synapses with a mean value 280% of that at nonCF-PC synapses (Supplementary Figure S3C). Overall, despite the limitations of the analysis, these results suggest that Shank2 may be more concentrated at CF-PC than nonCF-PC synapses whereas synGAP and Shank1 are selectively excluded from CF-PC relative to nonCF-PC synapses.

### NMDA receptor complexes are selectively distributed to extrasynaptic clusters in the granule cell layer

**Figure 7:**
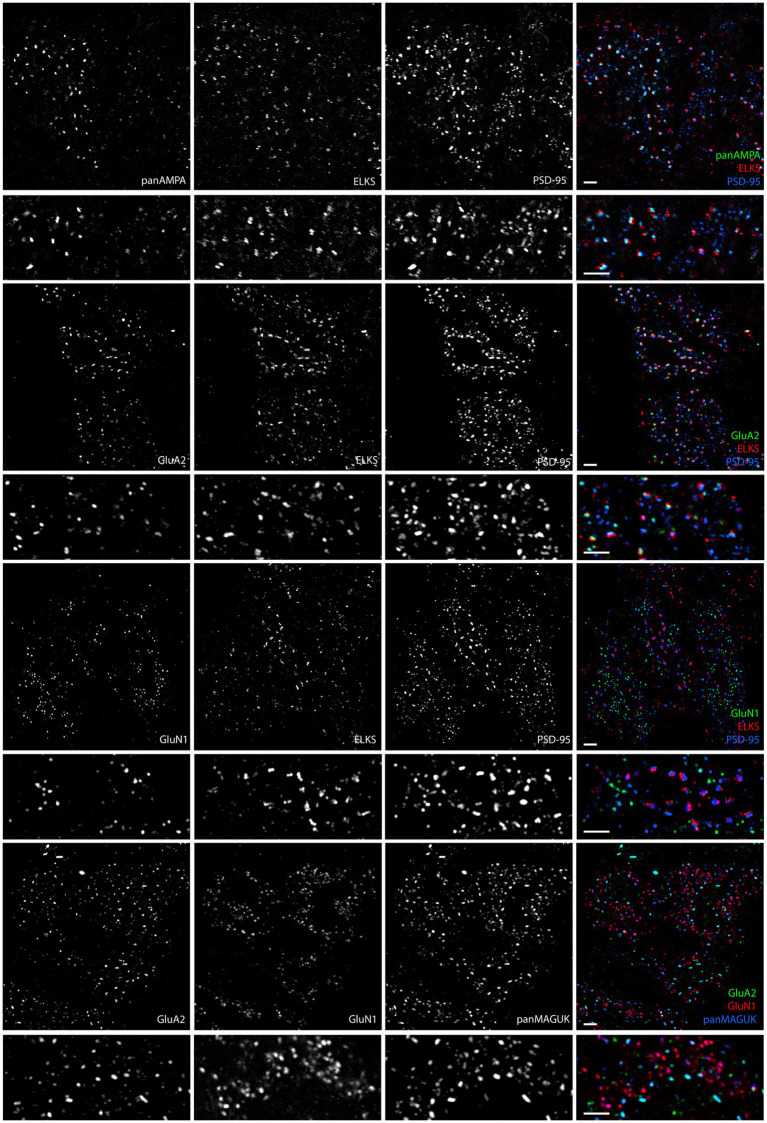
AMPA and NMDA receptors show differential synaptic versus extrasynaptic localization in glomeruli. Images of expanded mouse cerebellar sections taken in the granule cell layer showing panAMPA, GluA2, and GluN1 with ELKS and PSD-95. ELKS marks all synaptic sites in the glomeruli. PSD-95 and panMAGUK are in large clusters at excitatory synaptic sites adjacent to ELKS and in smaller clusters at extrasynaptic sites not associated with ELKS. While panAMPA and GluA2 selectively cluster at synaptic sites, GluN1 selectively clusters at extrasynaptic sites. As seen in the final panel, GluN1 and GluA2 labeled together with panMAGUK, the AMPA and NMDA receptor clusters show little to no overlap. Scale bars 1 μm biological scale, 3.66 μm expanded scale.

In the granule cell layer of the cerebellar cortex, we initially focused our efforts on localizing glutamate receptors within glomeruli using MAP processing. We used antibodies against panAMPA, the major AMPA receptor subunit GluA2, and the essential NMDA receptor subunit GluN1, along with the scaffold protein PSD-95 or panMAGUK corresponding to all PSD-95 family members. In an initial co-stain of GluA2 and GluN1, surprisingly, we observed largely distinct non-overlapping clusters for these AMPA and NMDA receptor subunits (Figure 7). To assess the relation of these AMPA and NMDA receptor clusters to synapses, we proceeded to co-stain for panAMPA, GluA2, or GluN1 together with PSD-95 and with the universal presynaptic component ELKS (Figure 7). panAMPA and GluA2 largely appeared to colocalize with PSD-95 and ELKS at synaptic sites, as expected. However, GluN1 rarely colocalized with ELKS but typically colocalized with smaller PSD-95 clusters lacking ELKS, indicating extrasynaptic clusters. To confirm the presence of extrasynaptic clusters of GluN1 and PSD-95, we co-stained together with ELKS and another universal presynaptic component RIM1/2 (Wang et al., 2016; Tan et al., 2022). Indeed, GluN1 clustered largely at extrasynaptic sites lacking ELKS and RIM1/2, while PSD-95 formed larger synaptic clusters with both ELKS and RIM1/2 and smaller extrasynaptic clusters lacking ELKS and RIM1/2 (Supplementary Figure S4). Thus, our findings support the localization of NMDA receptors to extrasynaptic sites (Petralia et al., 2002; Cathala et al., 2003) even though this has been controversial (Yamada et al., 2001; Abe et al., 2004; Ito, 2006).

**Figure 8:**
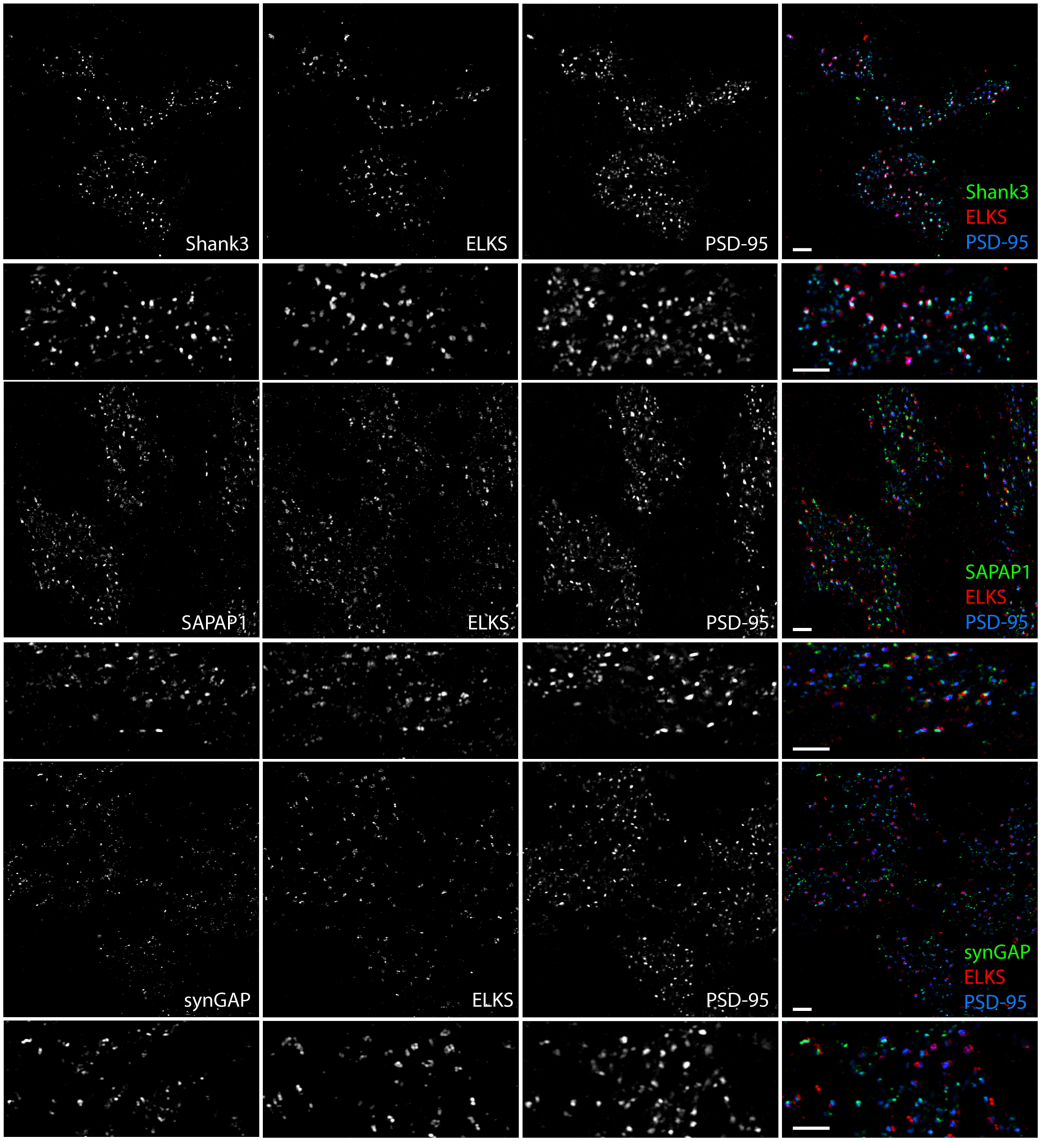
Synaptic versus extrasynaptic localization of scaffolding and signaling proteins in glomeruli. Images of expanded mouse cerebellar sections taken in the granule cell layer showing scaffolding and signaling proteins of interest with ELKS and PSD-95. While Shank3 and SAPAP1 localize to synaptic and extrasynaptic sites, SynGAP appears to preferentially localize to extrasynaptic sites. Scale bars 1 μm biological scale, 3.66 μm expanded scale.

To further investigate which synaptic proteins are expressed at synaptic versus extrasynaptic sites in the granule cell layer, we expanded the assays to include Shank3, SAPAP1, and synGAP, co-staining each with PSD-95 and ELKS. Shank1 and Shank2 are not expressed by granule cells (Kozareva et al., 2021) and we confirmed little or no signal by MAP (data not shown). Shank3 and SAPAP1 appeared to localize to both synaptic and extrasynaptic sites, whereas synGAP localized more to extrasynaptic sites (Figure 8).

**Figure 9:**
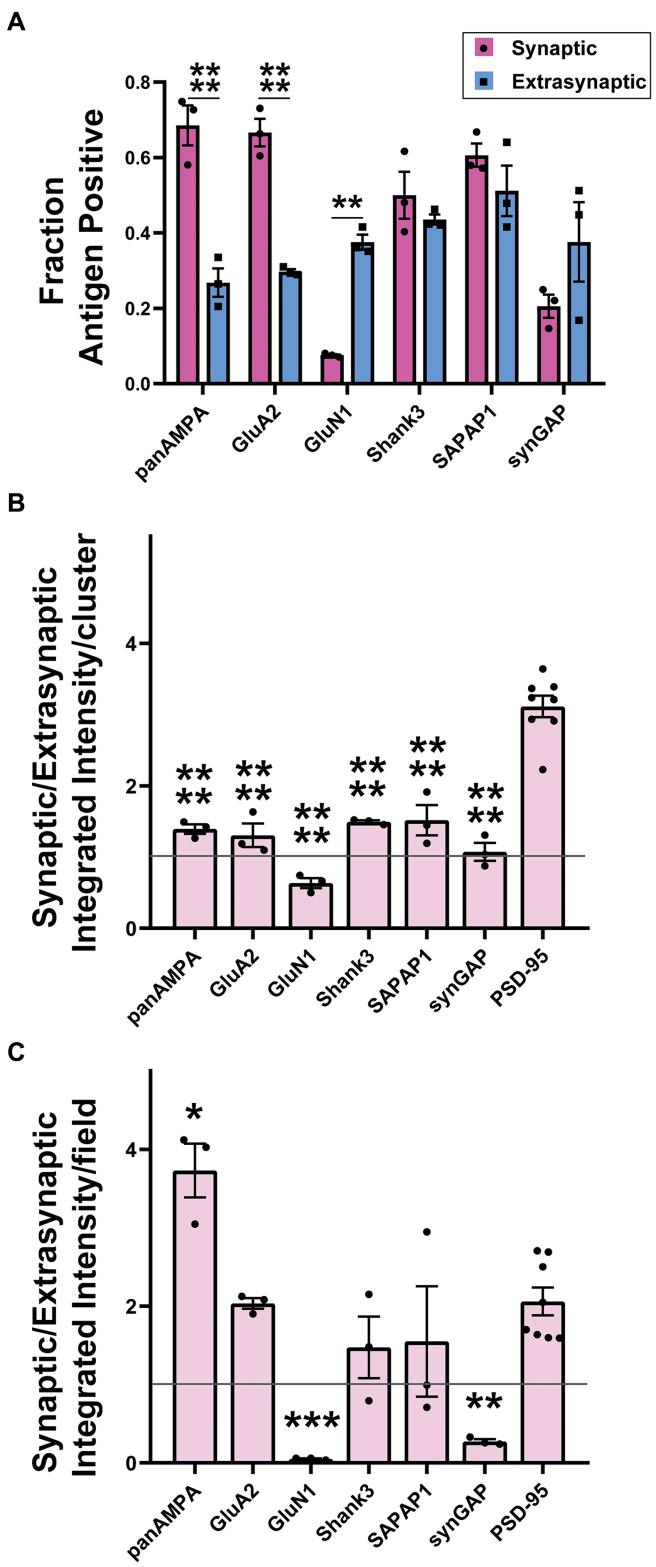
Quantitation of synaptic versus extrasynaptic localization in glomeruli. Brains were processed using the MAP procedure and each section was stained with an antigen of interest along with ELKS and PSD-95, as in Figures 7, 8. Two image stacks were taken per section, with two sections from each mouse, and a total of three mice. Values from each mouse were averaged (*n* = 3 mice). **(A)** Fraction of synaptic vs. extrasynaptic PSD-95 clusters positive for each antigen. 2-way ANOVA showed significant differences between antigens as well as between synaptic and extrasynaptic clusters: Antigen *p* < 0.0001, Synaptic vs. Extrasynaptic *p* = 0.009, interaction *p* < 0.0001. Sidak’s post-hoc comparisons showed that panAMPA (**** *p* < 0.0001) and GluA2 (**** *p* < 0.0001) were present at a significantly higher percentage of synaptic than extrasynaptic clusters, while GluN1 (** *p* = 0.001) was present at a significantly lower percentage of synaptic than extrasynaptic clusters, and the other antigens did not show a significant difference between synaptic and extrasynaptic (Shank3 *p* = 0.925, SAPAP1 *p* = 0.686, synGAP *p* = 0.108). **(B)** Ratio of integrated intensity per cluster at antigen-positive PSD-95-positive synaptic/extrasynaptic clusters for each antigen of interest in granule cell layer glomeruli. All ratios were > 1 except GluN1, which had a ratio of <1. One-way ANOVA showed significant differences between antigens: *p* < 0.0001. Dunnett’s post-hoc comparisons showed that all antigens (**** *p* < 0.0001) had a significantly lower ratio than PSD-95. **(C)** Ratio of integrated intensity per field at antigen positive PSD-95-positive synaptic/extrasynaptic clusters for each antigen of interest in granule cell layer glomeruli. One-way ANOVA showed significant differences between antigens: *p* < 0.0001. Dunnett’s post-hoc comparisons showed that when compared with the PSD-95 ratio, panAMPA (** *p* = 0.002) was significantly greater, while GluN1 (*** *p* = 0.0004) and synGAP (** *p* = 0.001) were significantly lesser, other antigens were not significantly different than PSD-95 (GluA2 *p* > 0.999, Shank3 *p* = 0.565, SAPAP1 *p* = 0.694). Each imaging field was 51.84 μm x 51.84 μm x 7.41 μm (X, Y and Z dimensions).

Quantitative measures in 3D confirmed these impressions, revealing significant differences among proteins and between synaptic and extrasynaptic clusters (Figure 9; Supplementary Figure S5). For this quantitation, synaptic sites were defined as being ELKS+ PSD-95+, while extrasynaptic sites were defined as being ELKS- PSD-95+. panAMPA and GluA2 were present at a significantly higher fraction of synaptic than extrasynaptic sites (Figure 9A). Combined with the measures of amount per cluster (Figure 9B; Supplementary Figure S5B), this resulted in a significant 3.7-fold higher total amount of panAMPA at synaptic relative to extrasynaptic sites and a trend toward a higher amount by 2.0-fold for GluA2 (Figure 9C). In contrast, GluN1 was present at a higher fraction of extrasynaptic than synaptic sites, with SynGAP showing a similar trend (Figure 9A), corresponding to a higher density of extrasynaptic than synaptic sites for both proteins (Supplementary Figure S5A). Combined with measures of amount per cluster (Figure 9B; Supplementary Figure S5B), this resulted in a significant 20.6-fold higher total amount of GluN1 at extrasynaptic relative to synaptic sites and a 3.7-fold difference for SynGAP (Figure 9C). PSD-95, used to define both synaptic and extrasynaptic clusters, showed the greatest difference in amount per cluster according to cluster type with 3.4-fold more at synaptic than extrasynaptic clusters (Figure 9B). Shank3 and SAPAP1 showed no difference between synaptic and extrasynaptic sites in most measures although the amount per cluster was higher for synaptic than extrasynaptic sites (Supplementary Figure S5B).

**Figure 10:**
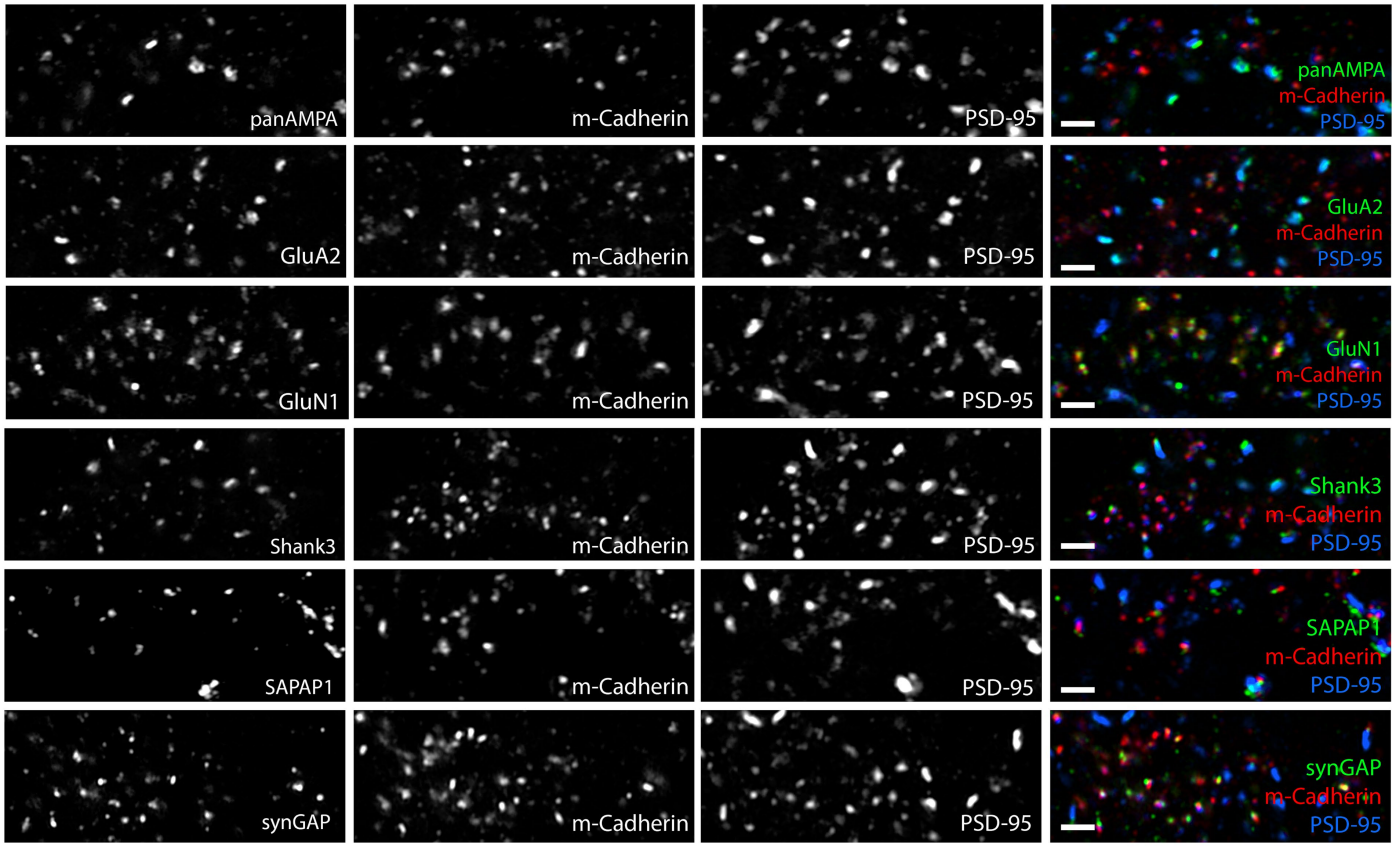
Glomerular NMDA receptor complexes are detected at adherens junctions. Images of expanded mouse cerebellar sections taken in the granule cell layer showing each antigen of interest along with M-cadherin and PSD-95. M-cadherin marks adherens junctions in the glomeruli. PSD-95 is detected at many adherens junctions as well as at larger, presumably synaptic, clusters. GluA2, panAMPA, Shank3 and SAPAP1 show minimal overlap with M-cadherin, consistent with their presence in synaptic clusters (as shown in Figures 8, 9). In contrast, GluN1 and SynGAP show strong colocalization with M-cadherin at adherens junctions. Scale bars 0.5 μm biological scale, 1.83 μm expanded scale.

Altogether, these results show that panAMPA and GluA2 cluster more strongly at synaptic sites, while GluN1 and synGAP cluster more strongly at extrasynaptic sites in cerebellar glomeruli. PSD-95 clusters at both sites but in larger amounts at synaptic sites, while Shank3 and SAPAP1 distribute more uniformly to synaptic and extrasynaptic clusters. A previous immunogold electron microscopy study found extrasynaptic NMDA receptors and PSD-95 localized to adherens junctions in cerebellar glomeruli (Petralia et al., 2002). Thus we co-stained our proteins of interest with M-cadherin, a specific marker of adherens junction in cerebellar glomeruli (Rose et al., 1995; Bahjaoui-Bouhaddi et al., 1997). While none of the other proteins showed any obvious association with M-cadherin, GluN1 and SynGAP as well as a number of the smaller PSD-95 clusters showed obvious colocalization with M-cadherin (Figure 10; Supplementary Figure S6). These findings confirm the localization of extrasynaptic NMDA receptors and PSD-95 to adherens junctions, and show that SynGAP is also present.

## Discussion

**Figure 11:**
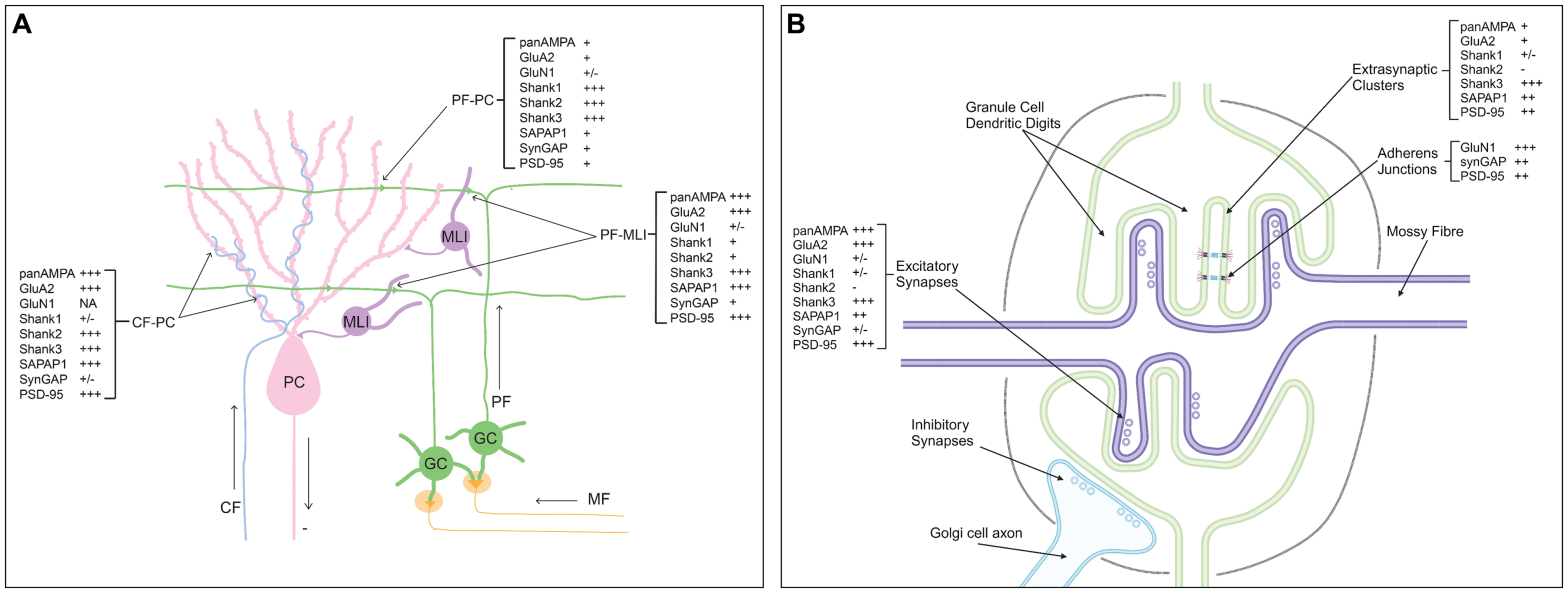
Summary of estimated relative abundance of synaptic components per synapse type in the cerebellar cortex. **(A)** Molecular layer. **(B)** Granule cell layer glomeruli. Created with BioRender.com.

Based on our proteomic mapping at single synapse resolution, we determined the relative abundance of multiple components at different synapse types in the cerebellar cortex; this is summarized in Figure 11. In the molecular layer, we confirmed the selective concentration of AMPA receptors and PSD-95 at CF-PC and PF-MLI synapses relative to PF-PC synapses and found a similar distribution for SAPAP1. Even among PF-PC synapses, AMPA receptors and SAPAP1 targeted selectively to the PSD-95+ subset. The three Shank family proteins showed a differential distribution, with Shank3 ubiquitously targeted to all synapses, Shank2 selective for PF-PC and CF-PC synapses, and Shank1 selective for PF-PC synapses. Thus, even within one cell type, PCs, proteins showed input-selective targeting, Shank1 to PF inputs and PSD-95, AMPA receptors and SAPAP1 to CF inputs. At synaptic sites in the granule cell layer, we confirmed selective localization of AMPA receptors as well as large clusters of PSD-95 and clusters of most synaptic proteins. However, perhaps our most surprising finding is the highly selective localization of NMDA receptors and SynGAP to extrasynaptic clusters in the granule cell layer, observed colocalizing with M-cadherin at adherens junctions.

Our data is consistent with studies on gene expression patterns (Lein et al., 2007; Kozareva et al., 2021), including higher expression of Shank1 and Shank2 and lower expression of PSD-95 in PCs than in MLIs. There are morphological differences in MLIs according to their position in the molecular layer, with basket cells prevalent proximally and stellate cells distally (Sotelo, 2015). However, we did not find significant differences in the synaptic proteome in the proximal versus distal molecular layer. This result is consistent with the more recent classification of MLIs into two molecularly and electrophysiologically distinct populations which are both distributed throughout the entire molecular layer (Kozareva et al., 2021; Hull and Regehr, 2022).

Many of our findings are not explained by simple differences in gene expression, revealing selective subcellular targeting to PF or CF inputs of PCs, and to synaptic versus extrasynaptic sites of GCs. Although the full signal transduction mechanism is not known, the levels of PSD-95 and AMPA receptors are upregulated at PF-PC synapses in the absence of GluD2, reducing the difference between PF-PC and CF-PC synapses (Yamasaki et al., 2011). It is not yet known whether SAPAP1 is regulated by a similar mechanism. SAPAP1 binds to PSD-95 (Naisbitt et al., 1997) and they showed similar subcellular targeting in both PCs and GCs. However, the observed subcellular targeting is not always explained in a simple way by known molecular interactions. SAPAP1 binds Shank1, Shank2, and Shank3 (Naisbitt et al., 1999) yet they did not show similar targeting, with SAPAP1 selective for CF inputs of PCs, Shank1 selective for PF inputs, and Shank2 and Shank3 targeting to both. In GCs, NMDA receptors and SynGAP were selectively targeted to extrasynaptic sites, yet they do not interact directly but rather both bind to PSD-95 (Kornau et al., 1995; Gamache et al., 2020) which was more concentrated at synaptic sites. Other interacting partners are likely involved in the specific subcellular targeting in both GCs and PCs, as well as local regulation by post-translational modifications. Both O-GlcNAcylation and phosphorylation of SynGAP by CaMKII reduce its binding to PSD-95 (Gamache et al., 2020; Lv et al., 2022), and other interactions may be similarly regulated.

The synapse-selective differences in AMPA receptor content in the molecular layer confirm previous immunogold electron microscopy studies (Masugi-Tokita et al., 2007; Yamasaki et al., 2011) and correspond to functional differences in efficacy of synaptic transmission (Barbour, 1993; Carter and Regehr, 2002; Foster et al., 2002). Functional consequences of the synapse-selective differences revealed here in the molecular layer for scaffolding proteins are currently harder to predict. The three Shank proteins share similar overall domains, mediating interactions with SAPAPs, Homer, cortactin, Rap and Ras, and self-multimerization (Sala et al., 2015; Lilja et al., 2017; Monteiro and Feng, 2017). Differences in fine structure among Shank1-3 may regulate interaction affinities. Furthermore, the three Shank genes undergo complex and differential region-selective alternative splicing and promoter usage, each generating protein products with only a subset of the above interaction domains and mediating different functions (Sala et al., 2015; Monteiro and Feng, 2017; Wan et al., 2022).

Perhaps our most surprising finding is the localization of NMDA receptor clusters including PSD-95 and SynGAP primarily to extrasynaptic sites in glomeruli of the granule cell layer. These extrasynaptic clusters may be fairly unique to cerebellar glomeruli. In our recent MAP studies in hippocampus and cortex, GluN1 and SynGAP clusters were essentially all tightly localized to synapses (Delhaye et al., 2024). The extrasynaptic cerebellar glomerular NMDA receptor complexes in the current study were distinct from AMPA receptor clusters associated with higher levels of PSD-95 as well as SAPAP1 and Shank3 at synaptic sites. Extrasynaptic NMDA receptors and PSD-95 were observed in cerebellar glomeruli previously by immunogold electron microscopy (Petralia et al., 2002), although this finding was controversial with other immunogold studies reporting a synaptic localization (Yamada et al., 2001; Abe et al., 2004). Perhaps the strongest evidence in favor of the extrasynaptic localization of NMDA receptors is functional data. At mature MF-GC synapses, quantal excitatory postsynaptic currents (EPSCs) activate only AMPA receptors while multiquantal EPSCs are required to also activate NMDA receptors (Cathala et al., 2003). These findings imply that NMDA receptors are located outside the synapse and are activated only by glutamate spillover (Szapiro and Barbour, 2007). The remarkable structure of glomeruli promotes signaling by spillover, as each MF release site has an additional ~7 release sites within 1 μm and a lack of intervening glia (Xu-Friedman and Regehr, 2003). AMPA receptors on GCs also show activation and even desensitization by glutamate spillover (DiGregorio et al., 2002; Xu-Friedman and Regehr, 2003). This glutamate spillover to both NMDA and AMPA receptors contributes to exceptionally high-frequency signaling in which rate and temporal coding convey sensory information (Delvendahl and Hallermann, 2016). During low-frequency MF inputs, these apparently extrasynaptic NMDA receptors contribute approximately half the MF-GC synaptic charge (Schwartz et al., 2012). These GC NMDA receptors are also required for potentiation of MF-GC synapses and vestibulo-cerebellar motor learning (Andreescu et al., 2011). Considering our data and a recent ultrastructural study (Nguyen et al., 2023), one may think of GC glomeruli as integrative signaling units composed of primarily synaptic AMPA receptors and extrasynaptic NMDA receptors on dendrites from ~15 GCs with each dendrite receiving inputs from ~10 release sites of a central MF bouton.

The observed selective localization of SynGAP with the extrasynaptic NMDA receptors and their association with adherens junctions within glomeruli raises intriguing possibilities for biochemical and structural regulation. These glomerular adherens junctions form between GC dendrites and are enriched in M-cadherin, α- and β-catenin, and actin filaments (Rose et al., 1995; Bahjaoui-Bouhaddi et al., 1997). NMDA receptor activation can regulate cadherin-mediated adhesion through multiple mechanisms including phosphorylation, proteolysis, endocytosis, and extracellular calcium levels (Tai et al., 2008). NMDA receptor activated cleavage of β-catenin can have far ranging consequences through triggering gene expression (Abe and Takeichi, 2007). SynGAP phosphorylation is also regulated by NMDA receptor activation and in turn regulates Ras and Rac, cofilin, and actin (Carlisle et al., 2008; Araki et al., 2015). These pathways have been studied at dendritic spine synapses where NMDA receptor complexes are typically located. It seems likely that such mechanisms could also operate at these adherens junctions to mediate activity regulation of glomerular structure impacting integrative signaling within glomeruli.

Our findings open up multiple directions for future research. Considering the ease of MAP relative to immunogold electron microscopy for proteomic mapping at single synapse resolution, expanding this study to additional receptors, scaffolding, and signaling proteins would be limited only by availability of suitable antibodies. By combining MAP with genetic labeling to identify cell types, it would be possible to assess synaptic differences between MLI subtypes and among GC layer interneurons, and perhaps determine whether the source of the MF and the participation of unipolar brush cells influences MF-GC composition. Another interesting question is whether synaptic composition is influenced by PC microzones, which differ in expression of aldolase C (zebrin II) and in physiological properties (Sillitoe et al., 2005; Hull and Regehr, 2022). Our findings also raise the intriguing questions of which additional signaling components are present in the adherens junction NMDA receptor complexes and how their activation may regulate glomerular structure and subsequent integrative signaling.

## Data availability statement

The original contributions presented in the study are included in the article/Supplementary material, further inquiries can be directed to the corresponding author.

## Ethics statement

The animal study was approved by University of British Columbia Animal Care Committee. The study was conducted in accordance with the local legislation and institutional requirements.

## Author contributions

KR: Conceptualization, Data curation, Formal analysis, Funding acquisition, Investigation, Methodology, Visualization, Writing – original draft, Writing – review & editing. MD: Conceptualization, Methodology, Writing – review & editing. AC: Conceptualization, Funding acquisition, Project administration, Supervision, Writing – original draft, Writing – review & editing.

## Supplementary material

SUPPLEMENTARY FIGURE S1 Quantitation of synaptic components at PF-PC and nonPF-PC synapses in the proximal and distal regions of the molecular layer. Brains were processed using the MAP procedure and each section was stained with an antigen of interest along with GluD2 and PSD-95, as in Figure 2. Two image stacks were taken from the proximal region of the molecular layer and two from the distal region per section, with two sections from each mouse, and a total of three mice. Values from each mouse were averaged (*n* = 3 mice). **(A)** Density/ 100μm^3^ (biological scale) of PF-PC and nonPF-PC synapses positive for each antigen of interest in the proximal and distal regions of the molecular layer. Total synapse densities for each class of synapse are indicated by the GluD2 values for PF-PC synapses and by the PSD-95 values for nonPF-PC synapses. 3-way ANOVA showed significance with antigen as well as PF-PC vs. nonPF-PC but not Proximal vs. Distal: Antigen *p* < 0.0001, Proximal vs. Distal *p* = 0.435, PF-PC vs. nonPF-PC *p* < 0.0001, Antigen x PF-PC vs. nonPF-PC *p* < 0.0001, Antigen x Proximal vs. Distal *p* = 0.526, Proximal vs. Distal x PF-PC vs. nonPF-PC *p* = 0.049, Antigen x Proximal vs. Distal x PF-PC vs. nonPF-PC *p* = 0.475. *p*-values for Tukey’s post-hoc comparisons can be found in Supplementary Table S1. **(B)** Integrated Intensity per synapse for antigen-positive PF-PC and nonPF-PC synapses in the proximal and distal regions of the molecular layer. Values were normalized to a mean of 1 for all synapses of all classes per antigen within an image stack. 3-way ANOVA showed significance with antigen as well as PF-PC vs. nonPF-PC but not Proximal vs. Distal: Antigen *p* < 0.0001, Proximal vs. Distal *p* = 0.981, PF-PC vs nonPF-PC *p* = 0.002, Antigen x PF-PC vs. nonPF-PC *p* < 0.0001, Antigen x Proximal vs. Distal *p* = 0.986, Proximal vs. Distal x PF-PC vs. nonPF-PC *p* = 0.221, Antigen x Proximal vs. Distal x PF-PC vs. nonPF-PC *p* = 0.339. *p*-values for Tukey’s post-hoc comparisons can be found in Supplementary Table S1.

SUPPLEMENTARY FIGURE S2 Quantitation of synaptic components at PSD-95- vs. PSD-95+ PF-PC synapses in the proximal and distal regions of the molecular layer. Brains were processed and imaged as in Figures 2, 3. PF-PC synapses were separated into categories according to the presence or absence of detectable PSD-95 and assessed for other synaptic components. **(A)** Density/ 100μm^3^ (biological scale) of PSD-95- and PSD-95+ PF-PC synapses positive for each antigen of interest at in the proximal and distal regions of the molecular layer. Total synapse densities for each class of synapse are indicated by the GluD2 values. 3-way ANOVA showed significance with antigen as well as PSD-95- vs. PSD-95+, but not Proximal vs. Distal: Antigen *p* < 0.0001, PSD-95- vs. PSD-95+ *p* < 0.0001, Proximal vs. Distal *p* = 0.560, Antigen x PSD-95- vs. PSD-95+ *p* < 0.0001, Antigen x Proximal vs. Distal *p* = 0.303, Proximal vs. Distal x PSD-95- vs. PSD-95+ *p* = 0.163, Antigen x Proximal vs. Distal x PSD-95- vs. PSD-95+ *p* = 0.910 (*n* = 3 mice). *p*-values for Tukey’s post-hoc comparisons can be found in Supplementary Table S1. **(B)** Integrated Intensity per synapse for antigen-positive PSD-95- and PSD-95+ PF-PC synapses in the proximal and distal regions of the molecular layer. Values were normalized to a mean of 1 for all synapses of all classes per antigen within an image stack.3-way ANOVA showed significance with antigen,PSD-95- vs. PSD-95+, and Proximal vs. Distal: Antigen *p* < 0.0001, PSD-95- vs. PSD-95+ *p* < 0.0001, Proximal vs. Distal *p* = 0.017, Antigen x PSD-95- vs. PSD-95+ *p* < 0.0001, Antigen x Proximal vs. Distal *p* = 0.919, Proximal vs. Distal x PSD-95- vs. PSD-95+ *p* = 0.704, Antigen x Proximal vs. Distal x PSD-95- vs. PSD-95+ *p* = 0.043 (*n* = 3 mice). *p*-values for Tukey’s post-hoc comparisons can be found in Supplementary Table S1.

SUPPLEMENTARY FIGURE S3 Quantitation of synaptic composition at CF-PC and nonCF-PC synapses. Brains were processed using the MAP procedure and each section was stained with an antigen of interest along with PSD-95 and VGluT2, as in Figure 5. Two image stacks were taken from the proximal region of the molecular layer and two from the distal region per section, with two sections from each mouse, and a total of three mice. Values from each mouse were averaged (*n* = 3 mice). **(A)** Density/100μm^3^ (biological scale) of antigen-positive PSD-95-positive CF-PC synapses in the molecular layer. Total synapse densities for each class of synapse are indicated by the PSD-95 values. One-way ANOVA showed that there were significant differences between antigens *p* = 0.025. Dunnett’s post-hoc comparisons showed that there were significant differences for Shank1 (* *p* = 0.043) and synGAP (** *p* = 0.0024) when compared with PSD-95, but no significance with other antigens (panAMPA *p* = 0.391, GluA2 *p* = 0.668, Shank2 *p* = 0.239, Shank3 *p* = 0.276, SAPAP1 *p* = 0.350) compared to PSD-95. **(B)** Density/100μm^3^ (biological scale) of antigen-positive PSD-95-positive nonCF-PC synapses in the molecular layer. One-way ANOVA showed that there were significant differences between antigens *p* < 0.0001. Dunnet’s post-hoc comparisons showed that there were significant differences for all antigens (panAMPA *** *p* < 0.001, Shank1 ** *p* = 0.006, Shank2 ** *p* = 0.002, Shank3 ** *p* = 0.003, SAPAP1 ** *p* = 0.001, synGAP **** *p* < 0.0001) except for GluA2 (*p* = 0.416) when compared with PSD-95. **(C)** Integrated Intensity per synapse for antigen-positive PSD-95-positive CF-PC and nonCF-PC synapses in the molecular layer. 2-way ANOVA did not show significance between antigens or CF-PC versus nonCF-PC: Antigen *p* = 0.150, CF-PC vs. nonCF-PC *p* = 0.055, interaction *p* = 0.060.

SUPPLEMENTARY FIGURE S4 Presence of extrasynaptic clusters of PSD-95 and GluN1 in the glomeruli of the granule cell layer. Images of expanded mouse cerebellar sections taken in the granule cell glomeruli. Co-staining of PSD-95 and GluN1 with two different synaptic markers, ELKS and RIM, showing the presence of PSD-95 and GluN1 clusters without synaptic markers. PSD-95 is present at both excitatory synapses, with ELKS and RIM, and at extrasynaptic sites, lacking ELKS and RIM, where GluN1 is present mainly at extrasynaptic sites. Scale bars 1 μm biological scale, 3.66 μm expanded scale.

SUPPLEMENTARY FIGURE S5 Quantitation of protein localization in glomeruli. Brains were processed using the MAP procedure and each section was stained with an antigen of interest along with ELKS and PSD-95, as in Figures 7, 8. Two image stacks were taken per section, with two sections from each mouse, and a total of three mice. Values from each mouse were averaged (*n* = 3 mice). **(A)** Density/100μm^3^ (biological scale) of synaptic and extrasynaptic antigen-positive PSD-95-positive clusters. 2-way ANOVA showed significant differences with antigen as well as synaptic vs. extrasynaptic: antigen *p* < 0.0001, synaptic vs. extrasynaptic *p* = 0.001, interaction *p* < 0.0001. Sidak’s post-hoc comparisons showed that GluN1 (* *p* = 0.016), SynGAP (* *p* = 0.018), and PSD-95 (**** *p* < 0.0001) were present at significantly more extrasynaptic clusters, while the other antigens did not show significant differences in synaptic vs. extrasynaptic sites (panAMPA *p* = 0.101, GluA2 *p* = 0.936, Shank3 *p* = 0.995, SAPAP1 *p* = 0.945). **(B)** Integrated intensity per cluster at PSD-95-positive synaptic and extrasynaptic sites for each antigen of interest in the granule cell layer glomeruli. 2-way ANOVA showed significant differences with antigen as well as synaptic vs. extrasynaptic: antigen *p* = 0.0001, synaptic vs. extrasynaptic *p* < 0.0001, interaction *p* < 0.0001. Sidak’s post-hoc comparisons showed that GluN1 (** *p* = 0.005) was significantly more intense at extrasynaptic clusters than synaptic clusters, and that Shank3 (** *p* = 0.004), SAPAP1 (* *p* = 0.014), and PSD-95 (**** *p* < 0.0001) were significantly more intense at synaptic clusters where panAMPA (*p* = 0.055), GluA2 (*p* = 0.2470) and synGAP (*p* > 0.999) were not significantly different at synaptic vs. extrasynaptic sites. **(C)** Integrated intensity per field at PSD-95-positive synaptic and extrasynaptic sites for each antigen of interest in the granule cell layer glomeruli. 2-way ANOVA showed significant differences with antigen and interaction but not with synaptic vs. extrasynaptic: antigen *p* < 0.0001, synaptic vs. extrasynaptic *p* = 0.227, interaction *p* < 0.0001. Sidak’s post-hoc comparisons showed that there was significantly more panAMPA (** *p* = 0.001) and PSD-95 (**** *p* < 0.0001) at synaptic sites than extrasynaptic sites, where there was significantly more GluN1 (** 0.002) and SynGAP (** 0.002) at extrasynaptic sites, and the remaining antigens did not show significant differences between synaptic and extrasynaptic sites (GluA2 *p* = 0.578, Shank3 *p* = 0.998, SAPAP1 *p* > 0.999).

SUPPLEMENTARY FIGURE S6 Glomerular NMDA receptor complexes are detected at adherens junctions. Images of expanded mouse cerebellar sections taken in the granule cell layer showing each antigen of interest along with M-cadherin and PSD-95. Larger fields of view are shown here than in Figure 10. Clusters of GluN1 and SynGAP are detected colocalizing with M-cadherin marking adherens junctions. Scale bars 1μm biological scale, 3.66 μm expanded scale.

SUPPLEMENTARY TABLE S1 Post-hoc comparison *p* values. The comparisons considered to be most meaningful are highlighted for the Tukey post-hoc comparisons following the 3-way ANOVA tests.
