# Supplementary figures and images for: Mapping proteomic composition of excitatory postsynaptic sites in the cerebellar cortex
Source: Front Mol Neurosci. 2024 May 9;17:1381534. doi: 10.3389/fnmol.2024.1381534 (PMC11111907; doi:10.3389/fnmol.2024.1381534)

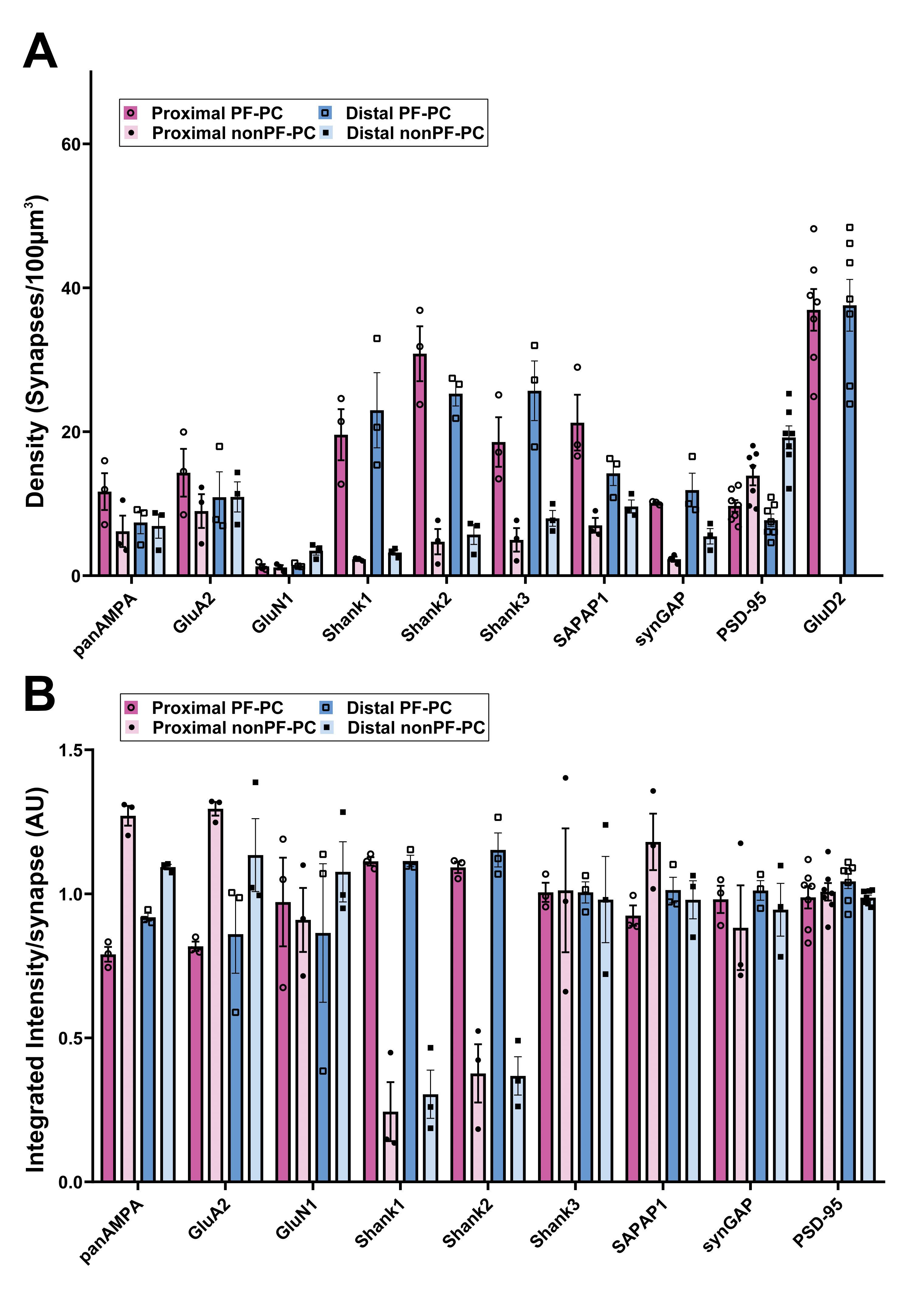

Supplement: SUPPLEMENTARY FIGURE S1 — Quantitation of synaptic components at PF-PC and nonPF-PC synapses in the proximal and distal regions of the molecular layer. Brains were processed using the MAP procedure and each section was stained with an antigen of interest along with GluD2 and PSD-95, as in Figure 2. Two image stacks were taken from the proximal region of the molecular layer and two from the distal region per section, with two sections from each mouse, and a total of three mice. Values from each mouse were averaged (n = 3 mice). (A) Density/ 100μm3 (biological scale) of PF-PC and nonPF-PC synapses positive for each antigen of interest in the proximal and distal regions of the molecular layer. Total synapse densities for each class of synapse are indicated by the GluD2 values for PF-PC synapses and by the PSD-95 values for nonPF-PC synapses. 3-way ANOVA showed significance with antigen as well as PF-PC vs. nonPF-PC but not Proximal vs. Distal: Antigen p < 0.0001, Proximal vs. Distal p = 0.435, PF-PC vs. nonPF-PC p < 0.0001, Antigen x PF-PC vs. nonPF-PC p < 0.0001, Antigen x Proximal vs. Distal p = 0.526, Proximal vs. Distal x PF-PC vs. nonPF-PC p = 0.049, Antigen x Proximal vs. Distal x PF-PC vs. nonPF-PC p = 0.475. p-values for Tukey’s post-hoc comparisons can be found in Supplementary Table S1. (B) Integrated Intensity per synapse for antigen-positive PF-PC and nonPF-PC synapses in the proximal and distal regions of the molecular layer. Values were normalized to a mean of 1 for all synapses of all classes per antigen within an image stack. 3-way ANOVA showed significance with antigen as well as PF-PC vs. nonPF-PC but not Proximal vs. Distal: Antigen p < 0.0001, Proximal vs. Distal p = 0.981, PF-PC vs nonPF-PC p = 0.002, Antigen x PF-PC vs. nonPF-PC p < 0.0001, Antigen x Proximal vs. Distal p = 0.986, Proximal vs. Distal x PF-PC vs. nonPF-PC p = 0.221, Antigen x Proximal vs. Distal x PF-PC vs. nonPF-PC p = 0.339. p-values for Tukey’s post-hoc comparisons can be found in Supplementary Table S1. [file Image_1.TIF]

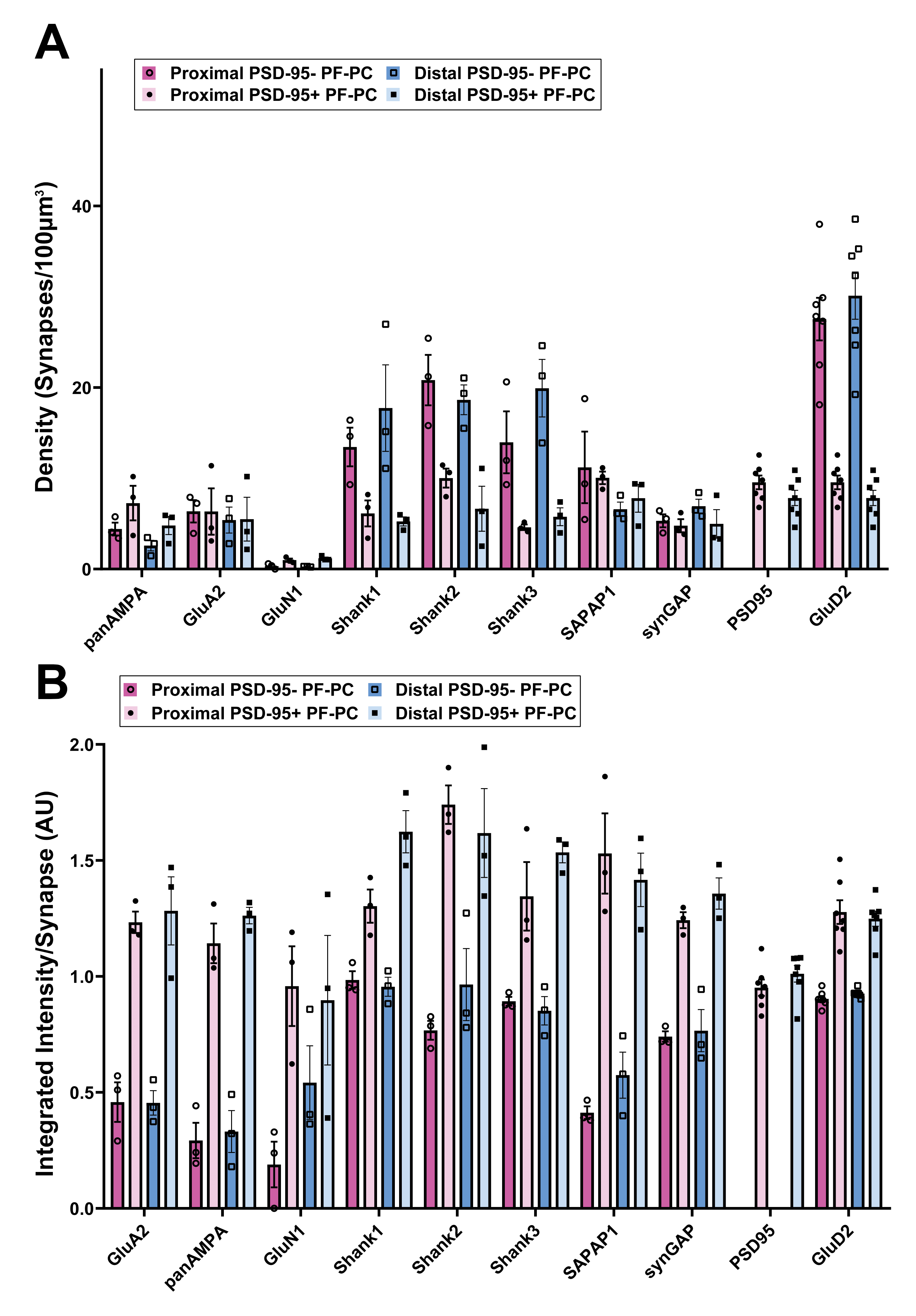

Supplement: SUPPLEMENTARY FIGURE S2 — Quantitation of synaptic components at PSD-95- vs. PSD-95+ PF-PC synapses in the proximal and distal regions of the molecular layer. Brains were processed and imaged as in Figures 2, 3. PF-PC synapses were separated into categories according to the presence or absence of detectable PSD-95 and assessed for other synaptic components. (A) Density/ 100μm3 (biological scale) of PSD-95- and PSD-95+ PF-PC synapses positive for each antigen of interest at in the proximal and distal regions of the molecular layer. Total synapse densities for each class of synapse are indicated by the GluD2 values. 3-way ANOVA showed significance with antigen as well as PSD-95- vs. PSD-95+, but not Proximal vs. Distal: Antigen p < 0.0001, PSD-95- vs. PSD-95+ p < 0.0001, Proximal vs. Distal p = 0.560, Antigen x PSD-95- vs. PSD-95+ p < 0.0001, Antigen x Proximal vs. Distal p = 0.303, Proximal vs. Distal x PSD-95- vs. PSD-95+ p = 0.163, Antigen x Proximal vs. Distal x PSD-95- vs. PSD-95+ p = 0.910 (n = 3 mice). p-values for Tukey’s post-hoc comparisons can be found in Supplementary Table S1. (B) Integrated Intensity per synapse for antigen-positive PSD-95- and PSD-95+ PF-PC synapses in the proximal and distal regions of the molecular layer. Values were normalized to a mean of 1 for all synapses of all classes per antigen within an image stack.3-way ANOVA showed significance with antigen,PSD-95- vs. PSD-95+, and Proximal vs. Distal: Antigen p < 0.0001, PSD-95- vs. PSD-95+ p < 0.0001, Proximal vs. Distal p = 0.017, Antigen x PSD-95- vs. PSD-95+ p < 0.0001, Antigen x Proximal vs. Distal p = 0.919, Proximal vs. Distal x PSD-95- vs. PSD-95+ p = 0.704, Antigen x Proximal vs. Distal x PSD-95- vs. PSD-95+ p = 0.043 (n = 3 mice). p-values for Tukey’s post-hoc comparisons can be found in Supplementary Table S1. [file Image_2.TIF]

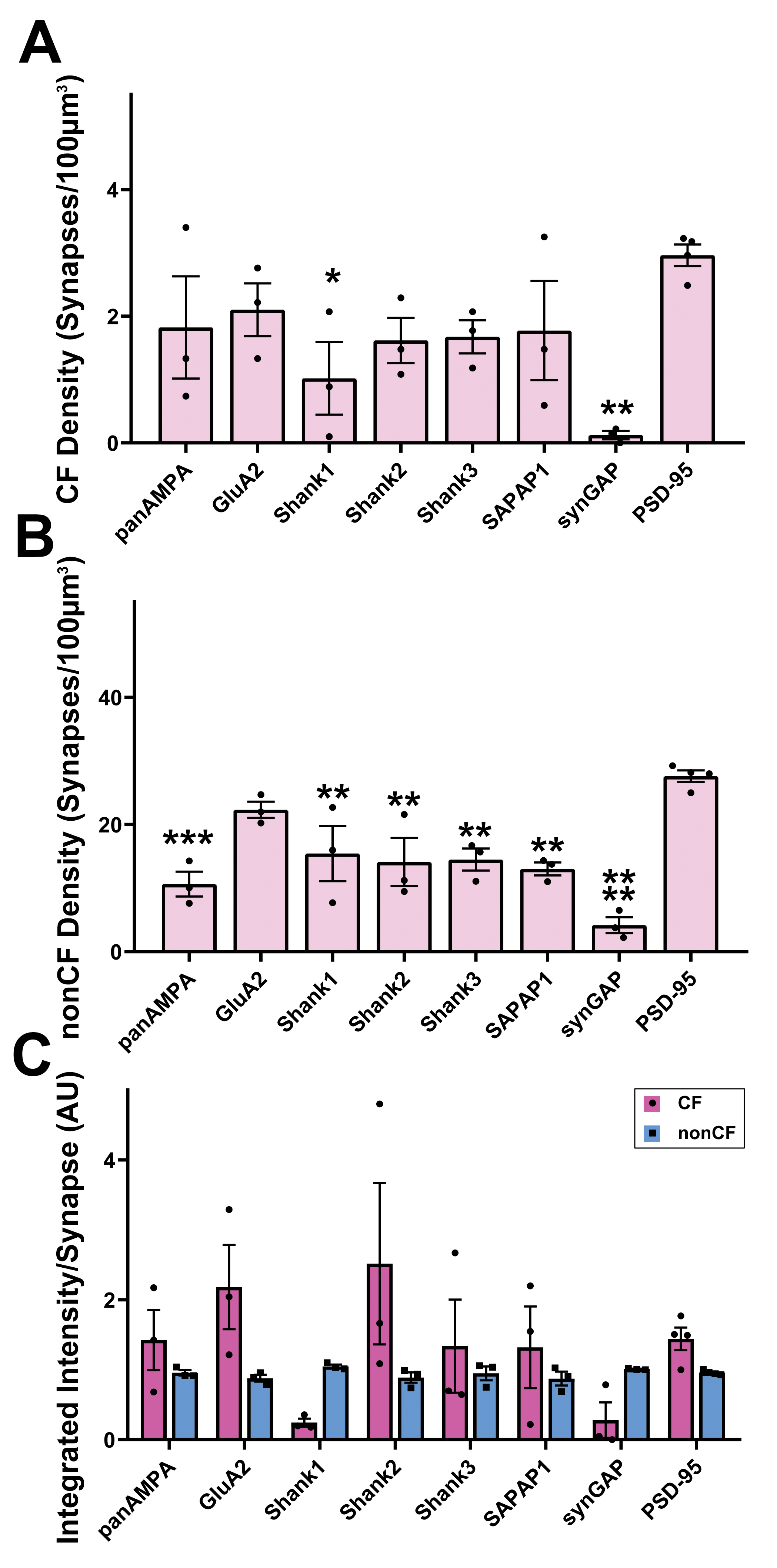

Supplement: SUPPLEMENTARY FIGURE S3 — Quantitation of synaptic composition at CF-PC and nonCF-PC synapses. Brains were processed using the MAP procedure and each section was stained with an antigen of interest along with PSD-95 and VGluT2, as in Figure 5. Two image stacks were taken from the proximal region of the molecular layer and two from the distal region per section, with two sections from each mouse, and a total of three mice. Values from each mouse were averaged (n = 3 mice). (A) Density/100μm3 (biological scale) of antigen-positive PSD-95-positive CF-PC synapses in the molecular layer. Total synapse densities for each class of synapse are indicated by the PSD-95 values. One-way ANOVA showed that there were significant differences between antigens p = 0.025. Dunnett’s post-hoc comparisons showed that there were significant differences for Shank1 (* p = 0.043) and synGAP (** p = 0.0024) when compared with PSD-95, but no significance with other antigens (panAMPA p = 0.391, GluA2 p = 0.668, Shank2 p = 0.239, Shank3 p = 0.276, SAPAP1 p = 0.350) compared to PSD-95. (B) Density/100μm3 (biological scale) of antigen-positive PSD-95-positive nonCF-PC synapses in the molecular layer. One-way ANOVA showed that there were significant differences between antigens p < 0.0001. Dunnet’s post-hoc comparisons showed that there were significant differences for all antigens (panAMPA *** p < 0.001, Shank1 ** p = 0.006, Shank2 ** p = 0.002, Shank3 ** p = 0.003, SAPAP1 ** p = 0.001, synGAP **** p < 0.0001) except for GluA2 (p = 0.416) when compared with PSD-95. (C) Integrated Intensity per synapse for antigen-positive PSD-95-positive CF-PC and nonCF-PC synapses in the molecular layer. 2-way ANOVA did not show significance between antigens or CF-PC versus nonCF-PC: Antigen p = 0.150, CF-PC vs. nonCF-PC p = 0.055, interaction p = 0.060. [file Image_3.TIF]

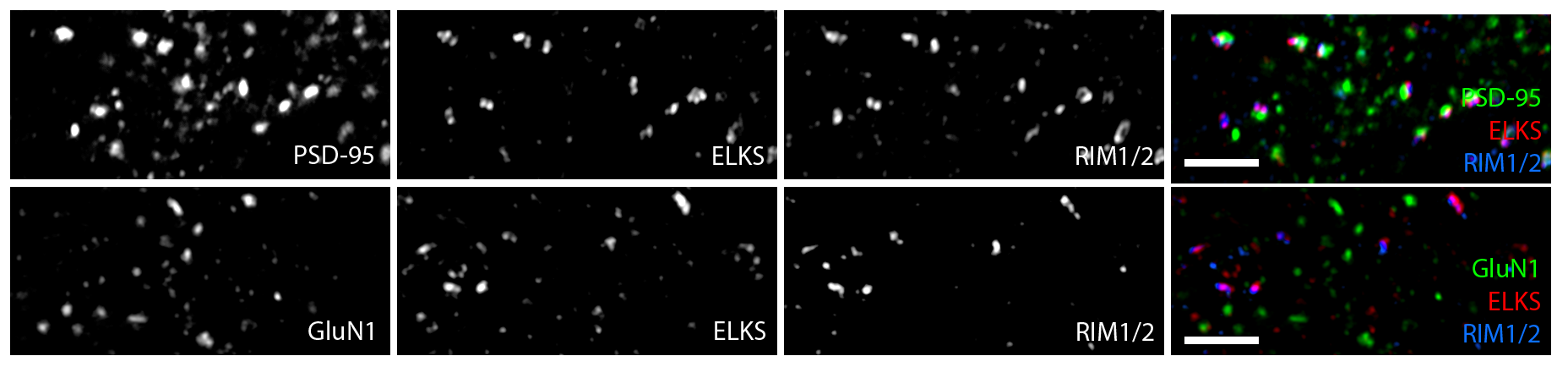

Supplement: SUPPLEMENTARY FIGURE S4 — Presence of extrasynaptic clusters of PSD-95 and GluN1 in the glomeruli of the granule cell layer. Images of expanded mouse cerebellar sections taken in the granule cell glomeruli. Co-staining of PSD-95 and GluN1 with two different synaptic markers, ELKS and RIM, showing the presence of PSD-95 and GluN1 clusters without synaptic markers. PSD-95 is present at both excitatory synapses, with ELKS and RIM, and at extrasynaptic sites, lacking ELKS and RIM, where GluN1 is present mainly at extrasynaptic sites. Scale bars 1 μm biological scale, 3.66 μm expanded scale. [file Image_4.TIF]

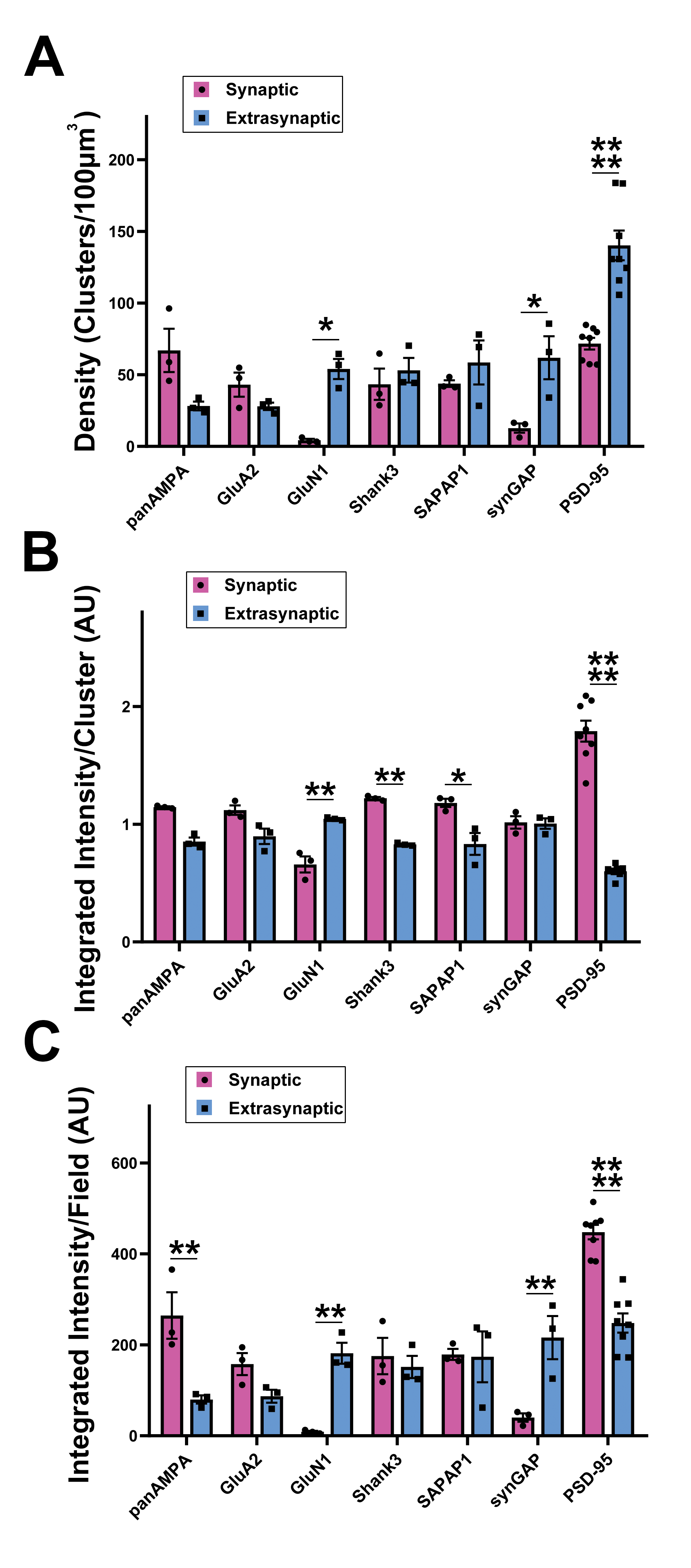

Supplement: SUPPLEMENTARY FIGURE S5 — Quantitation of protein localization in glomeruli. Brains were processed using the MAP procedure and each section was stained with an antigen of interest along with ELKS and PSD-95, as in Figures 7, 8. Two image stacks were taken per section, with two sections from each mouse, and a total of three mice. Values from each mouse were averaged (n = 3 mice). (A) Density/100μm3 (biological scale) of synaptic and extrasynaptic antigen-positive PSD-95-positive clusters. 2-way ANOVA showed significant differences with antigen as well as synaptic vs. extrasynaptic: antigen p < 0.0001, synaptic vs. extrasynaptic p = 0.001, interaction p < 0.0001. Sidak’s post-hoc comparisons showed that GluN1 (* p = 0.016), SynGAP (* p = 0.018), and PSD-95 (**** p < 0.0001) were present at significantly more extrasynaptic clusters, while the other antigens did not show significant differences in synaptic vs. extrasynaptic sites (panAMPA p = 0.101, GluA2 p = 0.936, Shank3 p = 0.995, SAPAP1 p = 0.945). (B) Integrated intensity per cluster at PSD-95-positive synaptic and extrasynaptic sites for each antigen of interest in the granule cell layer glomeruli. 2-way ANOVA showed significant differences with antigen as well as synaptic vs. extrasynaptic: antigen p = 0.0001, synaptic vs. extrasynaptic p < 0.0001, interaction p < 0.0001. Sidak’s post-hoc comparisons showed that GluN1 (** p = 0.005) was significantly more intense at extrasynaptic clusters than synaptic clusters, and that Shank3 (** p = 0.004), SAPAP1 (* p = 0.014), and PSD-95 (**** p < 0.0001) were significantly more intense at synaptic clusters where panAMPA (p = 0.055), GluA2 (p = 0.2470) and synGAP (p > 0.999) were not significantly different at synaptic vs. extrasynaptic sites. (C) Integrated intensity per field at PSD-95-positive synaptic and extrasynaptic sites for each antigen of interest in the granule cell layer glomeruli. 2-way ANOVA showed significant differences with antigen and interaction but not with synaptic vs. extrasynap [file Image_5.TIF]

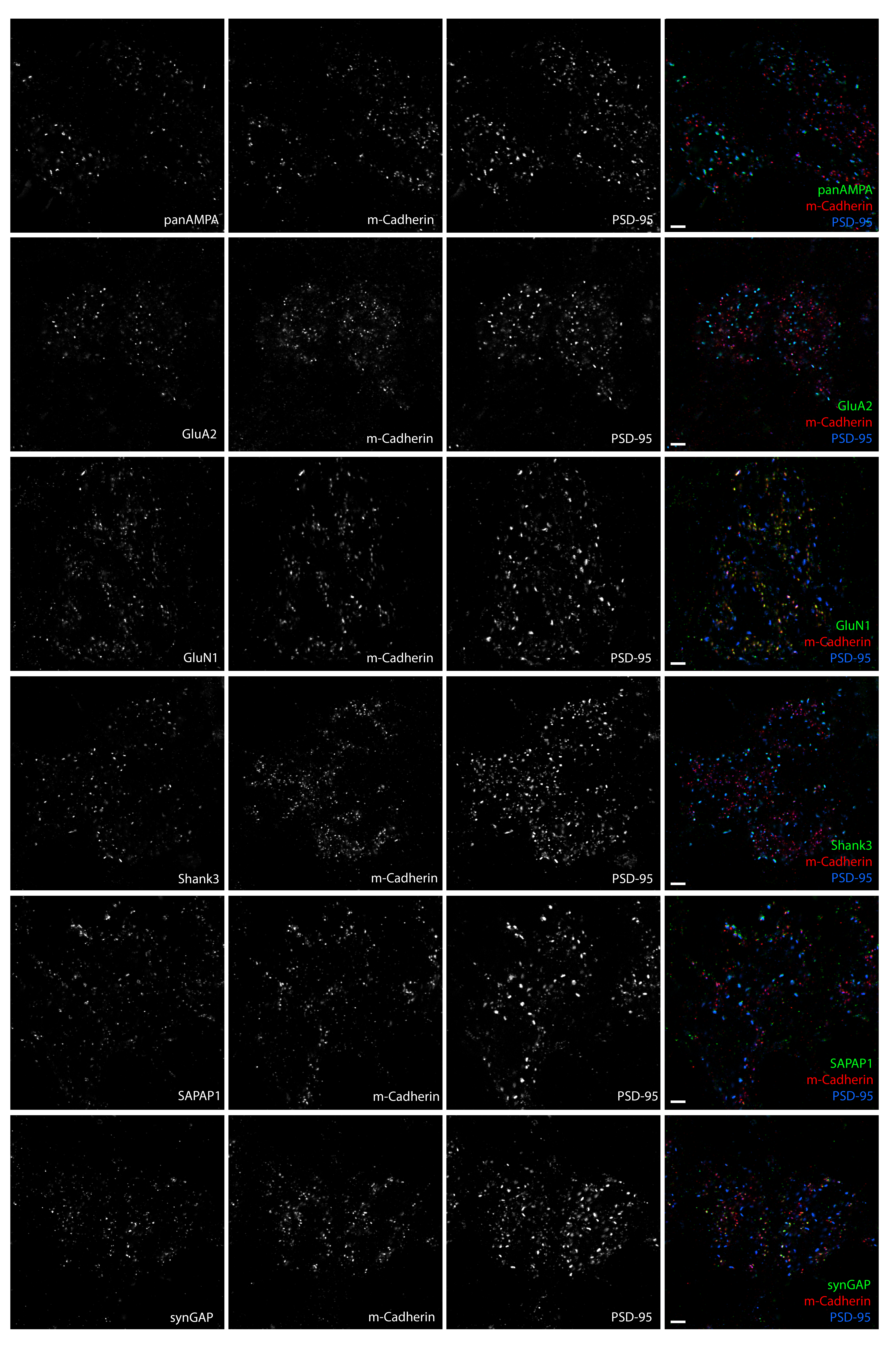

Supplement: SUPPLEMENTARY FIGURE S6 — Glomerular NMDA receptor complexes are detected at adherens junctions. Images of expanded mouse cerebellar sections taken in the granule cell layer showing each antigen of interest along with M-cadherin and PSD-95. Larger fields of view are shown here than in Figure 10. Clusters of GluN1 and SynGAP are detected colocalizing with M-cadherin marking adherens junctions. Scale bars 1μm biological scale, 3.66 μm expanded scale. [file Image_6.TIF]
